# Simulation of the Effects of Extracellular Calcium Changes Leads to a Novel Computational Model of Human Ventricular Action Potential With a Revised Calcium Handling

**DOI:** 10.3389/fphys.2020.00314

**Published:** 2020-04-15

**Authors:** Chiara Bartolucci, Elisa Passini, Jari Hyttinen, Michelangelo Paci, Stefano Severi

**Affiliations:** ^1^Computational Physiopathology Unit, Department of Electrical, Electronic and Information Engineering “Guglielmo Marconi”, University of Bologna, Cesena, Italy; ^2^Department of Computer Science, University of Oxford, Oxford, United Kingdom; ^3^BioMediTech, Faculty of Medicine and Health Technology, Tampere University, Tampere, Finland

**Keywords:** computational modeling, human ventricular action potential, population of models, calcium handling, extracellular concentrations

## Abstract

The importance of electrolyte concentrations for cardiac function is well established. Electrolyte variations can lead to arrhythmias onset, due to their important role in the action potential (AP) genesis and in maintaining cell homeostasis. However, most of the human AP computer models available in literature were developed with constant electrolyte concentrations, and fail to simulate physiological changes induced by electrolyte variations. This is especially true for Ca^2+^, even in the O’Hara-Rudy model (ORd), one of the most widely used models in cardiac electrophysiology. Therefore, the present work develops a new human ventricular model (BPS2020), based on ORd, able to simulate the inverse dependence of AP duration (APD) on extracellular Ca^2+^ concentration ([Ca^2+^]_o_), and APD rate dependence at 4 mM extracellular K^+^. The main changes needed with respect to ORd are: (i) an increased sensitivity of L-type Ca^2+^ current inactivation to [Ca^2+^]_o_; (ii) a single compartment description of the sarcoplasmic reticulum; iii) the replacement of Ca^2+^ release. BPS2020 is able to simulate the physiological APD-[Ca^2+^]_o_ relationship, while also retaining the well-reproduced properties of ORd (APD rate dependence, restitution, accommodation and current block effects). We also used BPS2020 to generate an experimentally-calibrated population of models to investigate: (i) the occurrence of repolarization abnormalities in response to hERG current block; (ii) the rate adaptation variability; (iii) the occurrence of alternans and delayed after-depolarizations at fast pacing. Our results indicate that we successfully developed an improved version of ORd, which can be used to investigate electrophysiological changes and pro-arrhythmic abnormalities induced by electrolyte variations and current block at multiple rates and at the population level.

## Introduction

In the last few decades, computational models have been increasingly used to study biological systems, due to the productive synergy between the *in silico* approach and the collection of experimental data, as well as to the improvement in computational resources. One of the most productive fields of application is cardiac physiology: modeling has provided physiological insights through the prediction of phenomena and mechanisms, later confirmed or disproved experimentally (e.g., Denis and Yoram, 2001; Carusi et al., 2012; Severi et al., 2014; Krogh-Madsen et al., 2016; Ni et al., 2018; Niederer et al., 2019).

One of the main causes of death all over the world is sudden cardiac death, which is correlated with the basic pro-arrhythmic mechanisms at the level of ion currents and single ventricular myocyte action potential (AP). In order to understand these mechanisms, and taking advantage of the increasing availability of specific data on ionic currents gathered from human cardiomyocytes, several mathematical models were developed to describe the biophysical mechanisms underlying the human ventricular AP. Currently, the “gold standard” for *in silico* human ventricular cellular electrophysiology is the O’Hara-Rudy (ORd) model (O’Hara et al., 2011). In the last few years, ORd was chosen as the consensus *in silico* model; it was used in multiple studies (e.g., Passini et al., 2015; Dutta et al., 2017b; Moreno et al., 2017) and selected for regulatory purposes (Dutta et al., 2017a). However, since mathematical models are reduced representations of the real biological systems, there is always room for improvements. In fact, the present work aims to propose a new and improved model of the human cardiac ventricular AP, starting from the ORd model.

When a new/updated model is proposed, two major points must be taken into consideration. Firstly, every model has its own validity, and what is the best model always depends on the aim of the investigation for which the model is used. Secondly, in order to claim that a model is actually an “improvement” of a previous one, it should be at least as effective as the previous model at reproducing the experimental data. This means that when a new specific question is addressed, none of the previous capabilities of the model should be lost. Keeping these points in mind, we developed a model oriented toward a specific research application: the investigation of the dependency of ventricular repolarization on extracellular electrolyte concentrations, particularly ionized Ca^2+^ concentration. This is especially meaningful, e.g., when considering new *in vitro* experiments with cardiomyocytes and ion concentrations set to values different than those considered for developing and tuning the model. At the same time, we sought to reproduce all the experimental protocols reported in the original ORd paper (O’Hara et al., 2011) and its further developments, such as the novel dynamic model for the rapid delayed K^+^ current (I_Kr_) that can capture drug-channel dynamic interactions (Grandi et al., 2010; Dutta et al., 2017a). Thus, we can also propose our new model as a general purpose and up-to-date model of human ventricular AP that can be used in a variety of applications, including drug trials.

It is well-known that extracellular Ca^2+^ concentration ([Ca^2+^]_o_) affects the cardiac AP: in fact, an increase of [Ca^2+^]_o_ shortens the AP while a decrease lengthens it. This has been observed in different species (guinea pig, dog, calf, and human) and different cell types (atrial, ventricular, and Purkinje fibers) (Temte and Davis, 1967; Kass, 1976; Leitch, 1996; Bai et al., 2005; Severi et al., 2009; Nagy et al., 2013; Supplementary Figure S1). Since both AP prolongation and shortening may lead to arrhythmia onset, the repolarization dependency on [Ca^2+^]_o_ may have important implications in all clinical contexts where electrolyte changes occur, such as haemodialysis therapy (Severi et al., 2008), pathological hypo/hypercalcemia, and head-down bed-rest experiments (Passini et al., 2013). Multiple ionic mechanisms are involved in the action potential duration (APD)-[Ca^2+^]_o_ dependence, which is why the phenomenon is not completely understood. Computational modeling may help elucidating it by analyzing the single ionic currents involved. However, most of the commonly used human ventricular AP models, which were developed using a single [Ca^2+^]_o_ value, are not able to reproduce the experimentally observed effects of [Ca^2+^]_o_ changes on APD. In fact, their APDs often display prolongation instead of shortening, and vice versa (as is the case with both the ORd (O’Hara et al., 2011) and the Grandi (Grandi et al., 2010, models).

When simulating the electrical activity of cardiac cells, it is also fundamental to set the same extracellular ionic concentrations used in experimental protocols, in order to properly compare *in silico* results and *in vitro* data. On the contrary, it would be incorrect to use the same concentrations when the analysis of *in vivo* pathophysiological mechanisms is the ultimate aim of simulation (Severi et al., 2009), since the *in vivo* and *in vitro* concentrations are different. The advantage of a model is that the extracellular ionic concentrations can be changed according to the study purpose, since they are parameters. However, this can be done only if the model correctly reproduces the main effects of such parameter changes. The non-physiological behavior of the ORd model (in response to changes of some extracellular ion concentrations) is one clear shortcoming of the model and of its usage for replicating specific *in vitro* and *in vivo* conditions. For this reason, we have modified the ORd model so that it can be used to simulate conditions where [Ca^2+^]_o_ changes and consequently also APD.

This work proposes a new human ventricular model (Bartolucci-Passini-Severi, BPS2020) which corrects the APD-[Ca^2+^]_o_ dependence of the ORd model, while still reproducing the large amount of experimental human data that was used for its development and validation. We paid particular attention in setting *in silico* the same extracellular concentrations used for *in vitro* experiments. Moreover, we constructed a population of models (Britton et al., 2013; Muszkiewicz et al., 2015; Paci et al., 2018a) to show that the BPS2020 model is a reliable baseline to reproduce the variability observed in experimental data from human undiseased heart (Britton et al., 2017). The population was also used to evaluate the ability of the model to reproduce pro-arrhythmic phenomena, such as early and delayed afterdepolarizations (EADs and DADs) and alternans, not systematically observed in experiments.

## Materials and Methods

### Model Development

#### Summary of the Model Development Strategy

The BPS2020 model was developed starting from the ORd model. Since one of the motivations of this study was to reproduce the physiological APD-[Ca^2+^]_o_ dependence, we first targeted the components of the model most likely contributing to this relationship. In particular, our earlier studies, based on the Ten Tusscher-Noble-Noble-Panfilov model (ten Tusscher et al., 2004), indicated that the L-Type Ca^2+^ current (I_CaL_) was primarily responsible for APD-[Ca^2+^]_o_ dependence. Two contrasting mechanisms are involved. When [Ca^2+^]_o_ is higher, it increases the I_CaL_ driving force which, by itself, would enhance the current density, and prolong the APD. On the other hand, a larger I_CaL_ also increases its Ca^2+^-dependent inactivation (CDI), which would reduce the current, and shorten the APD. Since the physiologically-observed outcome is APD shortening, we hypothesized that the increase in CDI must play the predominant role. Based on this hypothesis, the first change to the ORd model was a new I_CaL_ formulation (see section L-type Ca^2+^ Current), which strengthens the sensitivity of CDI to [Ca^2+^]_o_ compared to the ORd model. With this formulation, the increase in CDI induced by variations in [Ca^2+^]_o_ overcomes the effect of the increase in driving force, thus achieving the inverse APD-[Ca^2+^]_o_ relation.

After changing the I_CaL_ formulation, an overall adjustment of the Ca^2+^ handling was required, in order to maintain a correct APD rate dependence and a physiological Ca^2+^-induced Ca^2+^ release (CICR), as in the ORd model (see section Changes to the Ca^2+^ Handling). This was followed by an automated parameter optimization (similar to what was done in a previous study for a different model (Fabbri et al., 2017). The cost function was defined based on the consistency between the simulation results obtained with the BPS2020 model and the human experimental data presented in O’Hara et al. (2011) across a variety of protocols. We listed in Supplementary Table S3 the parameters that underwent the automatic optimization. At this stage, we also modified the extracellular K^+^ concentration ([K^+^]_o_) to match the one used in the experiments (see section Extracellular K^+^ Concentration and Liquid-Junction Potential Correction). More details on the optimization procedure and the cost function are available in Supplementary Material.

We reformulated a few currents/fluxes compared to the ORd model, as described below (see section L-type Ca^2+^ Current to Other Changes), while for others we only tuned the maximal conductances.

#### L-Type Ca^2+^ Current

**FIGURE 1:**
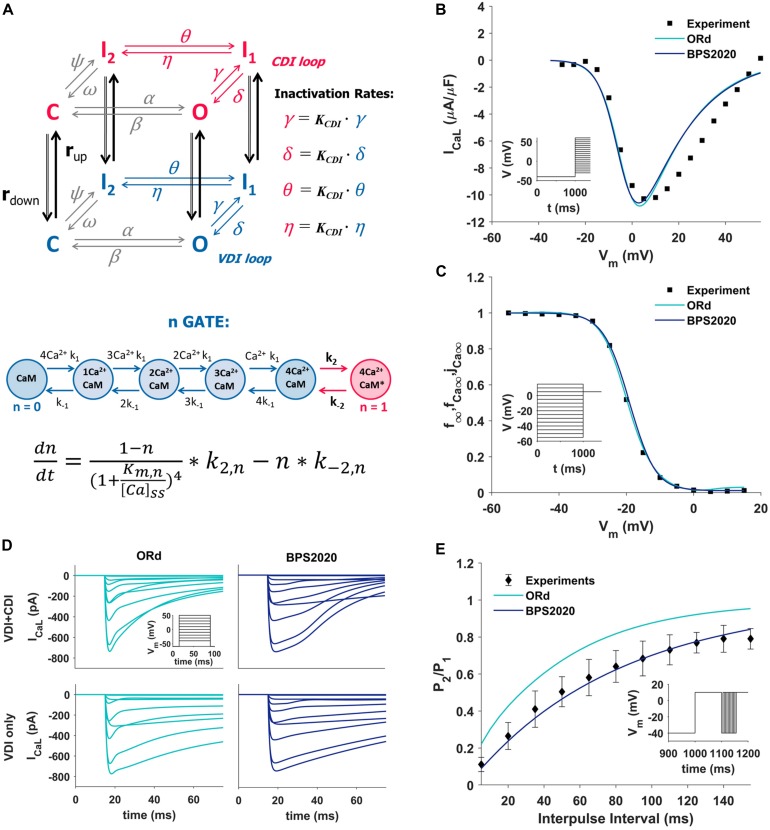
Summary of the design and validation of the new I_CaL_ model. **(A)** Schematic representation of the Markov model structure: voltage-dependent inactivation (VDI) and Ca^2+^-dependent inactivation (CDI) are represented as two separate loops, with four states each (C, closed; O, open; I_1_ and I_2_, inactivated). Inactivation rates in the CDI loop are K_CDI_ times faster than the corresponding rates in the VDI loop. VDI and CDI loops are interconnected by up/down rates (r_up_/r_down_), dependent on the n-gate, which directly depends on intracellular Ca^2+^ and its binding to Calmodulin (CaM), as shown in the equation at the bottom of the panel, modified from Decker et al. (2009); O’Hara et al. (2011), and Passini and Severi (2013). **(B,C)** Comparison of the simulated I_CaL_ I–V curve **(B)** and steady state inactivation curve **(C)** between ORd (light blue), BPS2020 (dark blue), and the experimental data from Magyar et al. (2000) (black squares). **(D)** Evaluation of the effect of CDI inactivation, by comparing VDI-only and VDI+CDI voltage clamp protocols for I_CaL_: both ORd (light blue, left) and BPS2020 (dark blue, right) show a faster inactivation when CDI is included, in agreement with experimental recordings with and without Ba^2+^ from O’Hara et al. (2011) (not shown). **(E)** Comparison of the recovery from inactivation between ORd (light blue), BPS2020 (dark blue), and the experimental data from Fülöp et al. (2004) (black diamonds), obtained using the P1/P2 protocol.

The I_CaL_ was completely revisited: its original Hodgkin-Huxley formulation was replaced by a new Markov model (Figure 1A, top panel), based on the structure proposed by Decker-Rudy for canine epicardial cells (Decker et al., 2009). This new formulation separates voltage dependent inactivation (VDI) and CDI in two loops, each consisting of the same four states: one closed (C), one open (O) and two inactivated (I_1_ and I_2_) states. Each loop includes four transitions: activation (from C to O), fast inactivation (from O to I_1_), slow inactivation (from I_1_ to I_2_) and recovery (from I_2_ to C). Activation and recovery rates (α/β and ψ/ω, respectively) are the same in the two loops; they were mainly derived from the ORd time constant and steady state values of the corresponding gating variables. In particular, the activation rates (α/β) were derived from the formulation of the *d* activation gate of ORd (see equations in Supplementary Material, section 3.4.3.4). The rates which govern the recovery from inactivation (ψ/ω), were derived from the steady state voltage-dependence of the *f* inactivation gate of ORd, and from a time constant tuned in order to fit the recovery from inactivation voltage-clamp data (Figure 1E). The γ/δ rates were based on the ORd inactivation gate (*f*) as well, but their range was limited to 0.2 to take into account the incomplete fast inactivation typical for I_CaL_ and the time constant was adjusted to reproduce voltage-clamp data. Finally, the rates η/θ modulate the slow inactivation from I_1_ to I_2_, and the equations were obtained by imposing the microscopic reversibility to the Markov model (see equations in Supplementary Material, section 3.4.3.7). Fast and slow inactivation rates (γ/δ and η/θ, respectively) in the CDI loop are K_CDI_ times faster than the ones in the VDI loop. This closely reflects the observation by Kim et al. (2004), that CDI works as a faster VDI, activated by elevated Ca^2+^. The CDI and VDI loops are connected by up/down rates (r_up_/r_down_), modulated by intracellular Ca^2+^ concentration, and controlled by the n gate. The K_CDI_ factor was initially set to 10, that is CDI was supposed to be 10 times faster than VDI. In fact, no published data are available which directly quantify this factor in the undiseased human ventricle. O’Hara et al. (2011), who determined the CDI time constants in an attempt to represent the shape and magnitude of their fractional remaining current (FRC) measurements, came to a formulation for the CDI fast time constant that is about (the ratio is voltage dependent there) six times faster than the VDI fast time constant. In our model, K_CDI_ was then automatically optimized, getting the final value of 9.

In the ORd model, the n gate represents the fraction of channels operating in CDI mode. It is the only I_CaL_ state variable directly dependent on Ca^2+^ concentration, and its formulation is based on the interaction between Ca^2+^ and the Calmodulin (CaM) bound to I_CaL_ channels (Figure 1A, bottom panel): when four Ca^2+^ ions bind to CaM (k_1_/k_–1_ rates), the Ca^2+^-CaM complex may activate CDI (k_2_/k_–2_ rates). In the BPS2020 model, the n gate controls r_up_/r_down_, thus modulating the fraction of channels in the VDI and CDI loop. Kinetics rates K_mn_ and k_–2n_ (see Supplementary Material) were modified compared to ORd, to increase the sensitivity of the n gate to Ca^2+^. All I_CaL_ equations are included in Supplementary Material.

The new I_CaL_ model was validated using four different I_CaL_ voltage-clamp protocols, the same used for the validation of the original ORd model. Simulation results obtained with the BPS2020 model were compared with the ones obtained with the ORd model, as well as with the corresponding experimental data.

The I–V and steady state inactivation curves were compared with data from Magyar et al. (2000) (Figures 1B,C). Results with BPS2020 and ORd are almost overlapped, and in agreement with the data. Simulations in VDI-only conditions (no CDI) are in agreement between BPS2020 and ORd: both models show a faster inactivation when both CDI and VDI are present (Figure 1D). These results are also in agreement with experimental recordings where Ba^2+^ was used as the current carrier, instead of Ca^2+^ (O’Hara et al., 2011). Recovery from inactivation was evaluated using a P_1_/P_2_ protocol, as in Fülöp et al. (2004): results with BPS2020 are closer to the experimental data than the ones obtained with ORd (Figure 1E). Our model effectively reproduces also the main features of FRC during the activation protocol for I_CaL_ (Supplementary Figure S4). In particular, FRC for CDI+VDI does not decrease monotonically with increasing voltage; rather, FRC curves appear to reproduce the experimentally observed “U shape” of the current inactivation. Moreover, VDI-alone is relatively weak, whereas CDI causes additional inactivation, whose maximal value is around 80%. On the other hand, some discrepancies with respect to the ORd data and model can be observed: at negative potentials CDI is very weak and at positive potentials, although the amount of CDI after 50 ms is very similar in the data and in the two models, CDI is a bit slower in ours, especially during the first milliseconds (e.g., it is still negligible after 5 ms).

#### Changes to the Ca^2+^ Handling

In addition to the I_CaL_ formulation, other changes in Ca^2+^-handling were needed to refine the BPS2020 model. The SERCA pump (Sarco-Endoplasmic Reticulum Ca^2+^ ATPase, J_up_) and the background Ca^2+^ currents were increased by factors of 3.13 and 4, respectively. The Ca^2+^ diffusion from subspace to bulk myoplasm was speeded up, by reducing the corresponding time constant. The sarcoplasmic reticulum (SR) was reduced to a single compartment, and its total volume was reduced by 5%, in agreement with other human computational models (Grandi et al., 2010; Paci et al., 2017) (see also Discussion, section APD Changes Induced by Extracellular Ca^2+^ Variations and Implications on Intracellular Ca^2+^ Handling). Consequently, Calsequestrin concentration ([CSQN]) was re-distributed in the whole SR: this reduced its concentration from 10 to 1 mM.

The SR Ca^2+^ release flux (J_rel_) via the ryanodine (RyR)-sensitive channels was replaced by the phenomenological formulation used by Paci et al. (2018b), and originally proposed by Koivumäki et al. (2011) for the human atrial myocyte. This formulation employs a Hodgkin-Huxley formalism, and it allows reproducing delayed after-depolarizations (DADs), which could not be reproduced with the original ORd formulation. Minor parameter changes were made to adapt the formulation to the different Ca^2+^ concentrations of BPS2020 (see Supplementary Material, section 3.4.4).

#### Extracellular K^+^ Concentration and Liquid-Junction Potential Correction

All the *in vitro* data published by O’Hara et al. (2011) were obtained with a [K^+^]_o_ = 4 mM, while all the ORd simulations were done with [K^+^]_o_ = 5.4 mM. In addition, as reported by O’Hara in the reader comments online (O’Hara et al., 2011), the *in vitro* experiments did not correct for the liquid-junction potential (LJP) at the electrode tip. In fact, in the I_K1_ experiments from O’Hara et al. (2011) the apparent reversal potential corresponded to [K^+^]_o_ = 5.4 mM despite the actual experimental concentration of 4 mM. The Nernst potential difference between the cases of 4 and 5.4 mM extracellular K^+^ is approximately +8 mV. Therefore, we introduced a positive shift equal to +8 mV (approximately corresponding to LJP) in the current equations built using the experimental data with [K^+^]_o_ = 4 mM. The affected equations are: the steady-state activation/inactivation and time constant of the transient outward K^+^ current (I_to_), I_K1_ formulation, and the steady state activation/deactivation and time constant of I_Ks_. In addition, we set [K^+^]_o_ = 4 mM in all the simulation protocols which are compared to these experimental data (e.g., rate dependence, restitution, drug block effects). The extracellular Na^+^ concentration ([Na^+^]_o_) was also changed from 140 to 144 mM.

#### Na^+^/Ca^2+^ Exchanger

The Na^+^/Ca^2+^ exchanger (I_NaCa_) formulation is the same as in the ORd model. We increased the maximum conductance by a factor of 2.4 upon automatic optimization.

#### Rapid Delayed Rectifier K^+^ Current (I_Kr_)

During the development of the BPS2020 model, a modification of the ORd model was published by Li et al. (2017) and Dutta et al. (2017a) within the Comprehensive in Vitro Proarrhythmia Assay (CiPA) initiative (Sager et al., 2014; Colatsky et al., 2016). This model was optimized for simulations of ion channel block and dynamic drug-channel interactions, and it used a new Markov model for I_Kr_ (Dutta et al., 2017a), that we decided to incorporate in the BPS2020 model. I_Kr_ maximum conductance was increased by 20% by the parameter optimization.

#### Inward Rectifier K^+^ Current (I_K1_)

The inward rectifier K^+^ current (I_K1_) maximum conductance was decreased by 29% and the steady state rectification slope was increased by 9%, following parameter optimization.

#### Slow Delayed Rectifier K^+^ Current (I_Ks_)

The slow delayed rectifier K^+^ current (I_Ks_) conductance was doubled, as in Dutta et al. (2017a). This was done to improve APD rate dependence under I_Kr_ channel block.

#### Late Na^+^ Current (I_NaL_)

The late Na^+^ current (I_NaL_) conductance was increased by a factor of 2.8, as in Dutta et al. (2017a).

#### Fast Na^+^ Current (I_NaF_)

The steady state inactivation and recovery from inactivation gates for I_NaF_ (h_ss_ and j_ss_) were modified as in Passini et al. (2015) (including also the CaMK phosphorylation pathway), to prevent propagation failure in hyperkalemia conditions. The time constants of inactivation gates were also modified, based on Dutta et al. (2017b). I_NaF_ conductance was reduced by 73% compared to its original value to preserve the peak current magnitude.

#### Na^+^/K^+^ ATPase Current (I_NaK_)

I_NaK_ maximum current was increased (twofold) to improve APD rate dependence (see Supplementary Figure S16). We consider this change reasonable, since no direct measurements of the Na^+^/K^+^ ATPase current (I_NaK_) in non-failing or healthy human ventricular cells are available, as also reported by O’Hara et al. (2011).

#### Other Changes

The current stimulus duration was set to 1 ms, with −53 μA/μF amplitude (twice the diastolic threshold), as in Passini et al. (2015). This allows to increase the maximum time step for integration to 1 ms.

### Single Cell Simulations

Simulations with the BPS2020 model were run using MATLAB R2018a (Mathworks Inc., Natick, MA, United States) on a Win 10 PC with an Intel Core i7. Numerical integration was performed with the Matlab function ode15s, a variable-step, variable-order solver, based on numerical differentiation formulas (Shampine and Reichelt, 1997). Pacing was maintained until steady state AP was reached (1,000 beats), and APD_X_ was measured once membrane voltage reached X% of the resting value (as in O’Hara et al., 2011). The code for running single cell simulations is available online at https://www.mcbeng.it/en/downloads/software/16-bps2020-model.html

### Population of Models

As in Britton et al. (2013) and Paci et al. (2017), a population of non-diseased AP models accounting for biological variability was constructed, by assuming that variability is mostly due to by cell-to-cell differences in ion channel densities rather than kinetics.

An initial random population of 5,000 human endocardial AP models was generated by sampling 11 parameters in the BPS2020 model, i.e., the maximum conductances, currents and fluxes of I_NaL_, I_NaF_, I_CaL_, I_to_, I_Kr_, I_Ks_, I_K1_, I_NaCa_, I_NaK_, J_rel_, and J_up_. The parameters were sampled in the range [20–200%] of their original values, using the Latin Hypercube Sampling (McKay et al., 1979).

The random population was then calibrated to select those models whose APs were in agreement with the *in vitro* non-diseased data (O’Hara et al., 2011) (summarized in Passini et al., 2017). The AP biomarkers used for the experimental calibration were: AP duration at 40, 50, and 90% of repolarization (APD_40_, APD_50_, and APD_90_), AP triangulation (Tri90-40, computed as the difference between APD_90_ and APD_40_), maximum upstroke velocity (dV/dt_max_), AP peak voltage (V_peak_) and resting potential (RMP). Each biomarker calibration range was built considering its minimum and maximum *in vitro* values. As an additional condition for calibration, we required that the models simulated correctly the inverse APD-[Ca^2+^]_o_ dependence, as detailed in section APD – [Ca^2+^]_o_ Dependence. Only models showing all the AP biomarkers within their *in vitro* ranges and an inverse APD-[Ca^2+^]_o_ dependence were accepted in the final calibrated population. The other models were discarded. As in Paci et al. (2017), we applied further additional conditions to avoid including models with clearly non-physiological intracellular ion concentrations. Only the models that had [Na^+^]_i_ in the range [5, 15] mM and the sarcoplasmic Ca^2+^ concentration ([Ca^2+^]_SR_) in [0, 10] mM (in steady state) were added to the calibrated population.

To assess the occurrence of delayed afterdepolarizations (DADs) we used the same protocol as in Li and Rudy (2011): starting from steady state each model in the population was paced for 1,500 beats at fast pacing (BCL = 300 ms, 3.3 Hz) and then for 1 long beat (BCL = 10,000 ms). DADs were identified visually.

We also used the population approach to study the occurrence of repolarization abnormalities, such as EADs and repolarization failures (RFs). The occurrence of repolarization abnormalities in the whole population was assessed after the administration of 0.1 μM dofetilide at a cycle length (CL) of 4,000 ms. The same extracellular concentration experimentally used by Guo et al. (2011) ([K^+^]_o_ = 5 mM, [Ca^2+^]_o_ = 2 mM, [Na^+^]_o_ = 137 mM) were used. The drug effect was simulated using the I_Kr_ drug binding values reported by Dutta et al. (2017a).

Finally, we challenged our *in silico* population with high pacing rate to assess variability in rate dependence and potential alternans occurrence: each model underwent increasing pacing rates, as in the 1998 work by Koller et al. (2017). The tested CLs were {600, 545, 500, 462, 429, 400, 375, 353, 333, 316, 300, 286, 273, 261, 250, 230} ms. For CLs greater than 300 ms models were paced for 30 s; otherwise for 15 s. The extracellular concentrations were set to [K^+^]_o_ = 5.4 mM, [Ca^2+^]_o_ = 1.2 mM and [Na^+^]_o_ = 140 mM. For this protocol, we performed an additional calibration step to filter the models displaying unphysiological concentrations as consequence of fast pacing. The upper bounds of [Na^+^]_i_ and [Ca^2+^]_SR_ were expanded by 50% with respect to the values used to calibrate the population in basal conditions. Therefore, models showing [Na^+^]_i_ and [Ca^2+^]_SR_ over 22.5 and 15 mM, respectively, were not considered for further analysis. Models were grouped in three classes: (i) adapting to the pacing rate increment without developing alternans (ADAPT), (ii) failing to adapt (ADAPT FAIL), and (iii) developing alternans (ALT).

To generate the population of models and running the DADs, dofetilide and alternans simulations, we used the Taito supercluster of CSC – IT Center for Science (Finland). We used Matlab R2017b on 50 CPU cores, using 3 GB/core memory. Generating the population took 21 h, while running DADs, dofetilide and alternans took 3, 15, and 8 h, respectively.

## Results

### APD-[Ca^2+^]_o_ Dependence

The challenge of reproducing the physiological APD-[Ca^2+^]_o_ dependence in computational cardiac models was first described by Severi et al. (2009). Most of the published models respond with AP prolongation to increases in [Ca^2+^]_o_, i.e., opposite of what observed *in vitro* and *in vivo* (Leitch, 1996; Severi et al., 2009; Nagy et al., 2013; Supplementary Figure S1), including ORd. The main goal of this work was to change the ORd model in order to achieve a physiological APD-[Ca^2+^]_o_ dependence, while preserving all its positive properties. This led to the development of the BPS2020 model.

**FIGURE 2:**
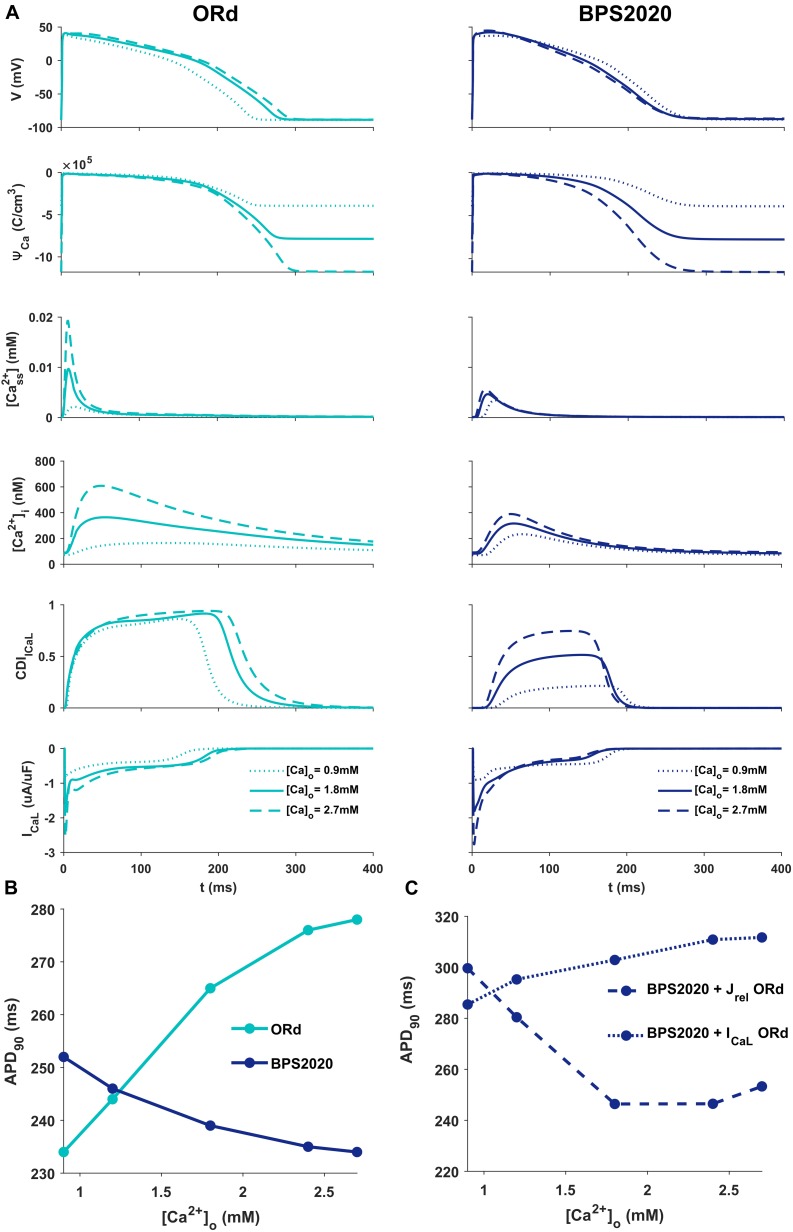
Comparison of ORd and BPS2020 behavior for [Ca^2+^]_o_ variations. **(A)** Simulated action potential (AP) for the original ORd and the BPS2020 models (left and right panels, respectively) for three different [Ca^2+^]_o_. In control conditions ([Ca^2+^]_o_ = 1.8 mM, solid lines), results with the two models are quite similar. However, when [Ca^2+^]_o_ increases ([Ca^2+^]_o_ = 2.7 mM, dashed lines) or decreases ([Ca^2+^]_o_ = 0.9 mM, dotted lines), they behave in two opposite ways. Only BPS2020 reproduces the inverse APD-[Ca^2+^]_o_ relationship observed experimentally. **(B)** APD-[Ca^2+^]_o_ relationship for ORd (light blue) vs. BPS2020 (dark blue). **(C)** Changes observed in the APD-[Ca^2+^]_o_ relationship of BPS2020 when restoring I_CaL_ (dotted line) or J_rel_ (dashed line) to the original ORd formulations.

Figure 2A illustrates the behavior of ORd (light blue) and BPS2020 (dark blue) for three different [Ca^2+^]_o_ with [K^+^]_o_ = 5.4 mM. When [Ca^2+^]_o_ is set to the control value (1.8 mM), simulations with BPS2020 and ORd produce very similar APs: APD_90_ at 1 Hz is 274 ms and 285 ms, for BPS2020 and ORd, respectively (Figure 2A, solid lines). This is in line with human experimental values of 277 ± 8 ms (O’Hara et al., 2011). However, when simulating different [Ca^2+^]_o_, an opposite behavior emerges. In both models, an increase in [Ca^2+^]_o_ causes an increase in driving force (*Ψ*_Ca_) and CDI (Figure 2A, dashed lines). However, in ORd the first mechanism prevails: I_CaL_ is enhanced and the consequence is AP prolongation. On the contrary, in BPS2020, the increase in *Ψ*_Ca_ is compensated by a much larger increase in CDI: this causes a faster I_CaL_ inactivation (see also Supplementary Figures S2, S3 for the states occupancy change over the course of action potential in the different conditions), leading to AP shortening. Interestingly, both models show larger intracellular Ca^2+^ concentration peaks, due to the increased I_CaL_, in the initial part of the AP (∼100 ms), before CDI starts playing a role. The same mechanisms are involved when considering a decrease in [Ca^2+^]_o_, which is causing AP shortening in ORd and prolongation in BPS2020 (Figure 2A, dotted lines). Therefore, the BPS2020 model is able to reproduce the physiological APD-[Ca^2+^]_o_ dependence (Figure 2B), not well simulated by ORd. An additional quantitative analysis of AP biomarkers dependence on extracellular electrolytes ([K^+^]_o_ and [Na^+^]_o_) was performed (see Supplementary Figure S14). The upper panels show that the model correctly reproduces the expected effects of hypo- and hyperkalemia: strong inverse relationship between APD and [K^+^]_o_ and strong positive dependence of the resting potential. The middle panels show that only a mild change (prolongation) of APD is caused by increasing [Na^+^]_o_ over a very large range. Finally, the bottom panels show that the inverse APD dependence on [Ca^2+^]_o_ is even larger for APD_70_ and APD_50_ than for APD_90_, which is consistent with the progressive increase in CDI with [Ca^2+^]_o_ and therefore the progressive reduction in I_CaL_, which exerts its predominant role in the phase 2 of the AP. On the other hand, the slight increase in AP peak is consistent with the progressive increase in I_CaL_ peak shown in Figure 2A.

To investigate if the inverse APD-[Ca^2+^]_o_ dependence is due to the new I_CaL_ formulation, or rather dependent on the new J_rel_ formulation, we ran two additional simulations with the BPS2020 model, separately restoring I_CaL_ and J_rel_ to the ORd formulation (Figure 2C). When restoring I_CaL_, the physiological APD-[Ca^2+^]_o_ dependence was lost (Figure 2C, dotted line), while it was preserved when restoring J_rel_ (Figure 2C, dashed line).

These results highlight the major role of I_CaL_ in controlling this phenomenon, and they support our initial hypothesis that the inverse APD-[Ca^2+^]_o_ dependence is mainly controlled by a stronger CDI.

### APD Rate Dependence

**FIGURE 3:**
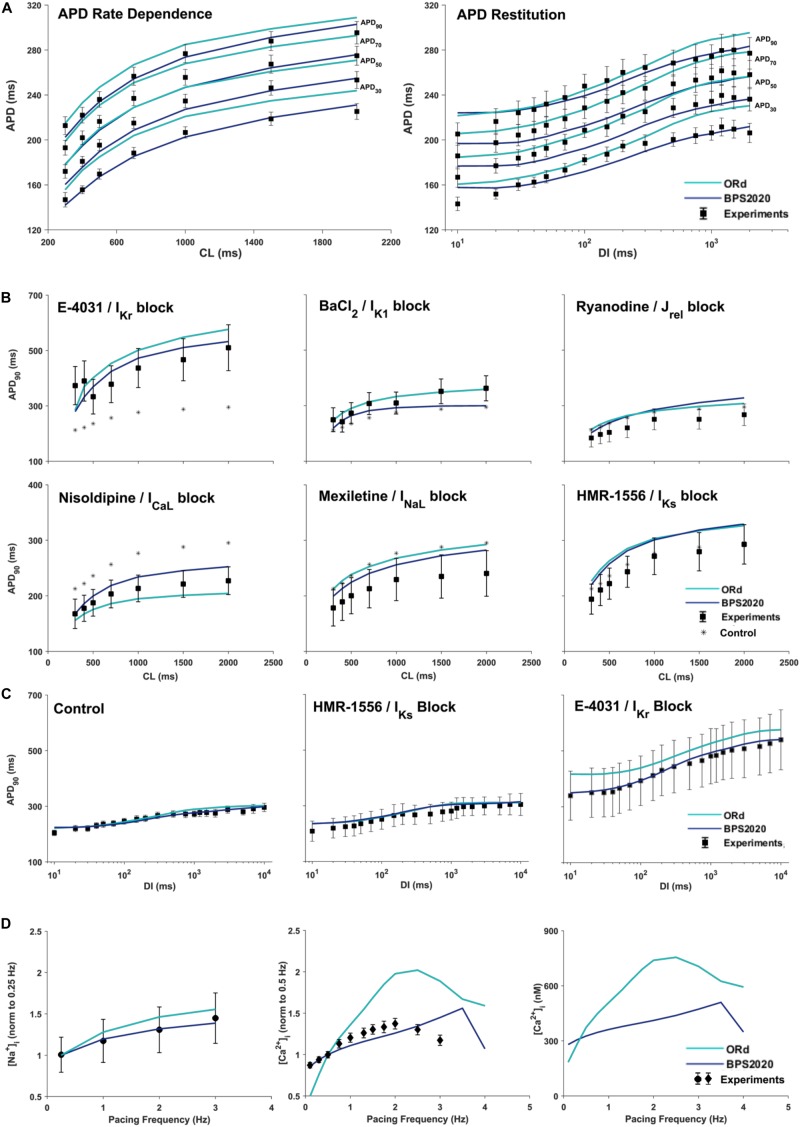
Rate dependence properties of BPS2020 vs. ORd. In all panels, simulation results for BPS2020 and ORd are shown in dark blue and light blue, respectively. Experimental data from O’Hara et al. (2011); Pieske et al. (2002) and Schmidt et al. (1998) are shown as black squares, black circles and black diamonds, respectively. **(A)** Steady state action potential duration (APD) rate dependence (CL – cycle length) and APD restitution obtained with the S_1_S_2_ protocol (DI – diastolic interval). APDs computed at 30, 50, 70, and 90% of repolarization are labeled on the right. **(B)** Steady state APD_90_ rate dependence changes induced by specific current blocks; stars are the APD_90_ values in control conditions. **(C)** APD_90_ restitution changes induced by specific current blocks. **(D)** [Na^+^]_i_ (left) and peak [Ca^2+^]_i_ (middle and right) vs. pacing frequency.

Figure 3A compares simulation results obtained with BPS2020 and ORd for the APD rate dependence (left panel) and restitution protocols (right panel), computed as in O’Hara et al. (2011). APD rate dependence was computed at steady state for different CLs, while APD restitution was computed using the S_1_/S_2_ protocol, i.e., steady state at CL = 1,000 ms (S_1_), followed by a single S2 extra-systolic stimulus, delivered at various diastolic intervals (DIs). APDs at 30, 50, 70, and 90% of repolarization were compared against the same human experimental data used for ORd validation (O’Hara et al., 2011). Simulations were run with [K^+^]_o_ = 4 mM, as in the experiments. BPS2020 is in qualitative and quantitative agreement with the experimental data, both for APD rate dependence and restitution. ORd model is in qualitative agreement with the experimental data, but simulated AP are overall longer and slightly different from those published in O’Hara et al. (2011), due to the changes in [K^+^]_o_.

Figures 3B,C illustrate how APD rate dependence (Figure 3B) and restitution (Figure 3C) vary in presence of specific channel blockers. Again, simulations were run with [K^+^]_o_ = 4 mM for both BPS2020 and ORd, as in the experiments (O’Hara et al., 2011). Some current block percentages were refined compared to the ones used in O’Hara et al. (2011), based on current literature: for E-4031 1 μM and HMR-1556 1 μM, we simulated 70% I_Kr_ block and 90% I_Ks_ block, respectively, as in O’Hara et al. (2011); for nisoldipine 1 μM we simulated 100% I_CaL_ block, as in Walsh et al. (2007); for BaCl_2_ 100 μM we simulated 84% I_K1_ block as in Biliczki et al. (2002); for mexiletine 1 μM we simulated its multichannel action, i.e., 54% I_NaL_ block, 20% I_CaL_ block and 9% I_Kr_ block, as in Dutta et al. (2017a); for ryanodine 5 μM we simulated 30% J_rel_ block. Indeed, according to literature data (Zucchi and Ronca-Testoni, 1997; Xu et al., 1998; Thomas and Williams, 2012), 5 μM ryanodine seems a too low concentration to induce 90% block of J_rel_, as assumed in O’Hara et al. (2011). E.g., ryanodine at 1 nM–10 μM, in bilayer studies, causes the RyR to open permanently to a sub conductance level (∼half of the fully open state, Rousseau et al., 1987). Moreover, ryanodine effects also depend on time of exposure. As reported in Zucchi and Ronca-Testoni, 1997), the incubation time required for inhibition was 5–10 min with 500 mM ryanodine and 1 h with 1 mM ryanodine. With 5 μM the time required is likely even longer. However, unfortunately, O’Hara-Rudy did not give details about the time of exposure (O’Hara et al., 2011). Thus, the quantification of RyR block by ryanodine is very tricky, but it is likely that 5 μM produces a block level definitely lower than 90%.

### Intracellular Na^+^ and Ca^2+^ Rate Dependence

Figure 3D shows how different pacing frequencies affect [Na^+^]_i_ (left panel) and peak [Ca^2+^]_i_ (middle and right panels). We compared simulation results against the human experimental data by Pieske et al. (2002) for [Na^+^]_i_ and Schmidt et al. (1998) for [Ca^2+^]_i_, normalized to 0.25 Hz and 0.5 Hz, respectively. To facilitate a quantitative comparison between BPS2020 and ORd, simulated [Ca^2+^]_i_ data are also shown as non-normalized (Figure 3D right panel). Extracellular concentrations were set to the experimental values: [Na^+^]_o_ = 152 mM, [K^+^]_o_ = 3.6 mM and [Ca^2+^]_o_ = 1.8 mM, for [Na^+^]_i_; [Na^+^]_o_ = 146 mM, [K^+^]_o_ = 5.9 mM and [Ca^2+^]_o_ = 2.5 mM for [Ca^2+^]_i_. BPS2020 and ORd were first paced at 0.5 Hz until steady-state, and then paced additional 100 beats for each frequency. Results with BPS2020 qualitatively and quantitatively reproduce the experimental data for [Na^+^]_i_, and they qualitatively reproduce the ones for [Ca^2+^]_i_, even though the decrease in peak [Ca^2+^]_i_ is observed at a faster pacing rate (4 Hz) than *in vitro* (2.5 Hz, simulations vs. experiments). Simulations with the ORd model are different from those shown in O’Hara et al. (2011), possibly due to differences in the extracellular concentrations or simulation protocol, since full simulation details are not provided in O’Hara et al. (2011).

In addition, since CaMK is important for controlling rate dependence of Ca^2+^ cycling, its effects are reported in Supplementary Figure S5, the results were in agreement with ORd. Supplementary Figure S6 shows the Ca^2+^ concentration in the SR of the BPS2020 model compared to the Ca^2+^ concentration in the SR junctional and network compartments in the ORd model.

### APD Accommodation

**FIGURE 4:**
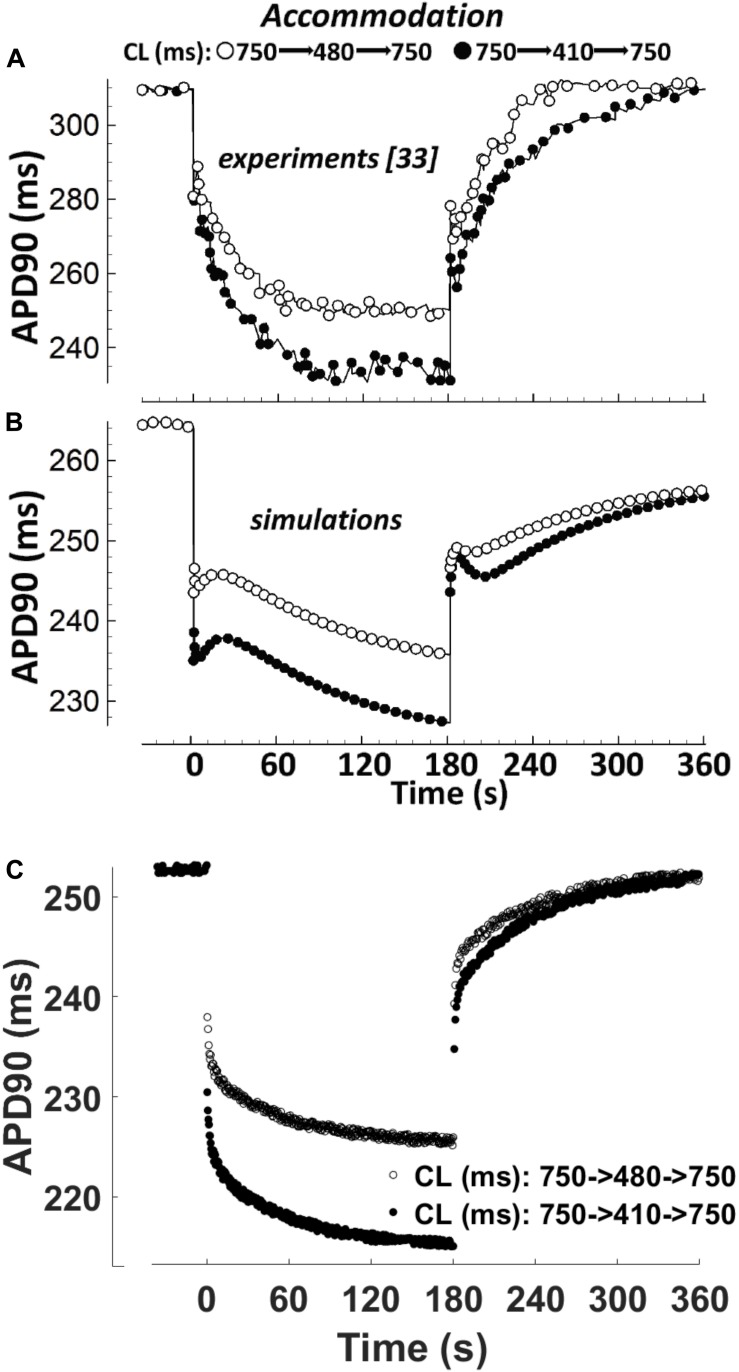
APD_90_ accommodation. At *t* = 0 s, the pacing cycle length (CL) is abruptly reduced from 750 to 480 ms (black circles) or 410 ms (white circles). At *t* = 180 s, the CL is abruptly increased to its original value. **(A)** Action potential duration (APD) accommodation measured experimentally by Franz et al. (1988). **(B)** APD accommodation simulated with the ORd model. **(C)** APD accommodation with the BPS2020 model. **(A,B)** Are adapted from O’Hara et al. (2011).

APD accommodation, i.e., the time course of APD response to abrupt changes in pacing rate, was measured in human patients by Franz et al. (1988), and highlighted as a marker of arrhythmic risk in a simulation study by Pueyo et al. (2010), performed with the Ten Tusscher-Panfilov model (ten Tusscher, 2006). Figure 4 shows a comparison between the *in vivo* experimental recordings (Figure 4A), and the corresponding simulation results for ORd and BPS2020 (Figures 4B,C, respectively). The BPS2020 model shows a very good qualitative agreement with the experiments, and the APD accommodation curve has a more physiological shape compared to ORd.

### Transmural Heterogeneity

To reproduce transmural heterogeneity, we created two additional versions of the BPS2020 model (EPI and M cells), by scaling specific ionic current conductances in the endocardial version (ENDO) of the model described until now. Changes were based on the same mRNA and protein expression data used for the ORd model (O’Hara et al., 2011), and full details are included in Supplementary Table S1. Simulation results for the three different cell types are shown in Supplementary Figure S10, compared with the human experimental data reported by Drouin et al. (1995) and Glukhov et al. (2010). Experimental data have been rescaled with respect to the ORd ENDO APD_90_ data (O’Hara et al., 2011), to facilitate the comparison in terms of EPI/ENDO and M/ENDO ratios.

### Population of Models (EADs, DADs, and Alternans)

**FIGURE 5:**
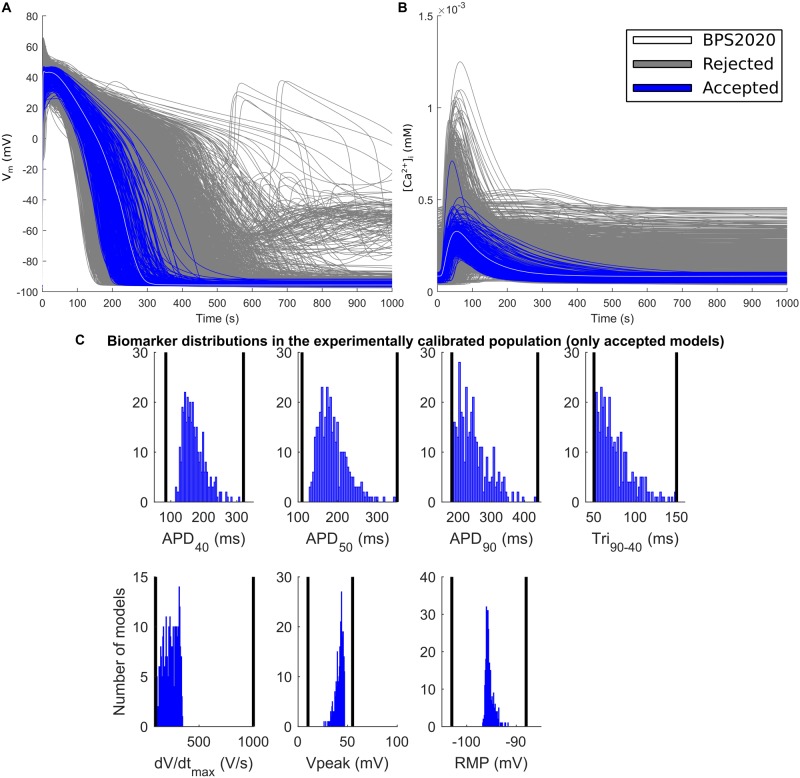
Experimentally calibrated population. **(A)** Action potentials and **(B)** Ca^2+^ transients with the BPS2020 model (baseline, white traces), the experimentally calibrated population (blue traces, representing 342 APs) and the rejected models (gray traces). **(C)** Biomarker distributions in the experimentally calibrated population. The black vertical lines are the experimental boundaries Passini et al. (2017) used to calibrate the population. APD, AP duration at the % specified repolarization; Tri_90–40_, APD_90_-APD_40_; dV/dt_max_, maximum upstroke velocity; Vpeak, AP peak voltage; RMP, resting membrane potential.

The experimental calibration selected a population of 342 models out of the initial 5,000; their APs and Ca^2+^ transients are shown in Figures 5A,B while the AP biomarker distributions are reported in Figure 5C. This population was used to test the occurrence of repolarization abnormalities and alternans.

**FIGURE 6:**
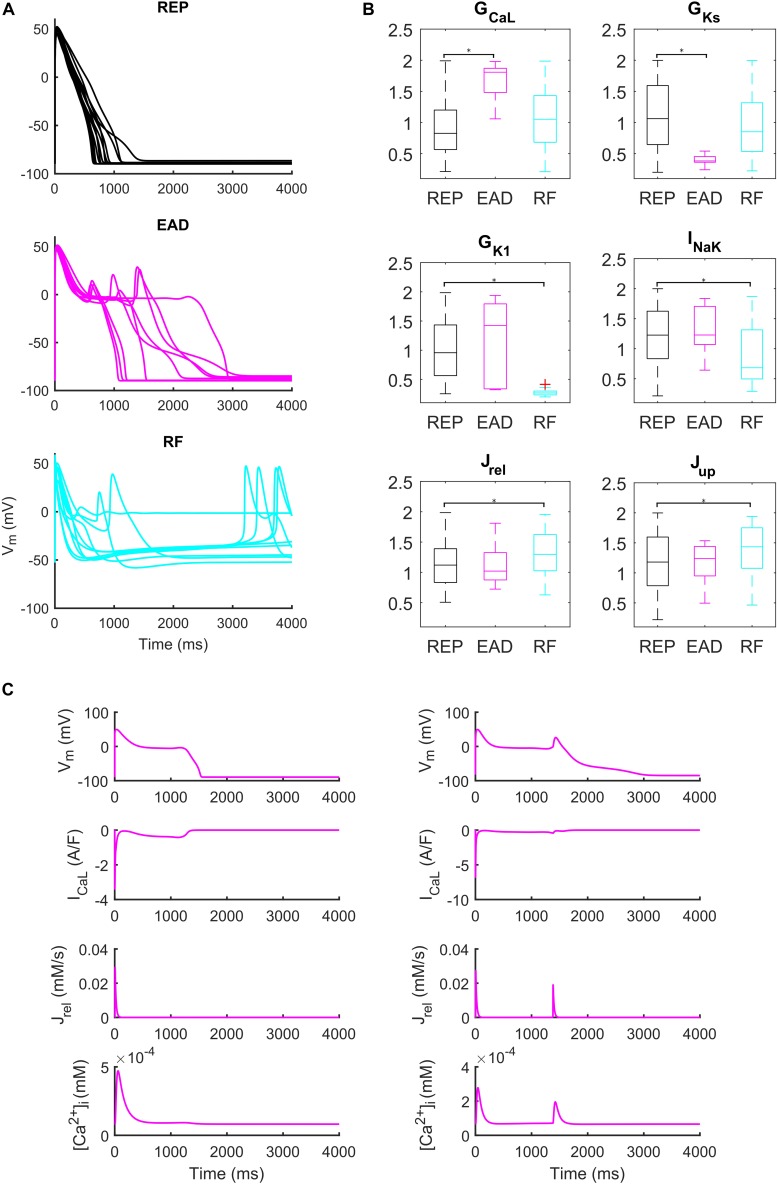
Repolarization abnormalities – EADs. **(A)** Examples of different responses to dofetilide (0.1 μM), CL = 4,000 ms (Guo et al., 2011). Top: repolarizing models (black, REP); middle: models developing early afterdepolarizations (magenta, EAD) and bottom panel failing to repolarize (cyan, RF). **(B)** Distribution of the scaling factors which show statistically significant differences between the three categories: models repolarizing (REP, black), models developing EADs (EAD, magenta) and models failing to repolarize (RF, cyan) (*p < 0.05). Red crosses represent outliers. **(C)** EAD induced by I_CaL_ reactivation (left) and EAD induced by Ca^2+^ release from SR (right).

Administration of 0.1 μM dofetilide induced early afterdepolarizations (EADs) in 9 models, repolarization failure (RF) in 40 models and simple AP prolongation with no pro-arrhythmic events (REP) in 293 models. Illustrative AP traces for the three groups are reported in Figure 6A, while significant (*p* < 0.05) differences in the sampled parameters among the REP, EAD, and RF classes are reported in Figure 6B. Interestingly, models belonging to the EAD class showed smaller I_Ks_, denoting smaller repolarization reserve, compared to the REP models. The RF models showed smaller I_K1_ than the REP ones, in line with the I_K1_ role of stabilizing the resting potential. Models developing EADs showed significantly higher I_CaL_ than the other classes; in fact, it is well-known that one of the mechanisms leading to EADs is actually I_CaL_ reactivation during phase three of the AP (Zeng and Rudy, 1995; Figure 6B, and expanded version in Supplementary Figure S13). Another outward current affecting AP repolarization is I_NaK_, therefore, if it is lower (as in the RF group), it provides less contribution to AP repolarization. However, in Priori and Corr (1990), another mechanism for the occurrence of EADs was highlighted: they concluded that Ca^2+^ release from SR activates an inward current (e.g., I_NaCa_) that carries the depolarizing charge for EADs (Figure 6C). These mechanisms underlying EAD generation are in agreement with previous simulation studies (Passini et al., 2015, 2017).

**FIGURE 7:**
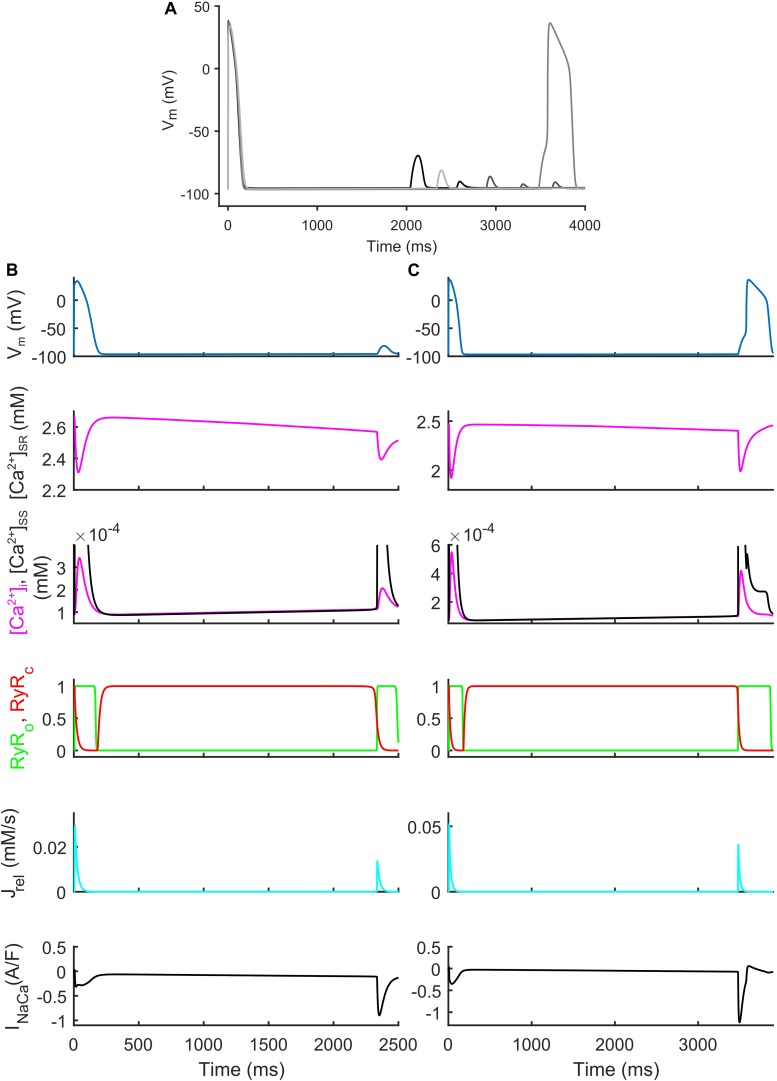
Repolarization abnormalities – DADs. **(A)** Illustrative action potential (AP) traces for four models that produced delayed afterdepolarizations (DADs). **(B)** Illustrative model producing a DAD. **(C)** Example of DAD degenerating into an anticipated spontaneous AP. In both models the leakage J_leak_ from the overloaded SR increased the Ca^2+^ concentrations in cytosol ([Ca^2+^]_i_) and subspace ([Ca^2+^]_SS_). The RyR-sensitive channels sensed the increased [Ca^2+^]_SS_ and triggered a spontaneous SR Ca^2+^ release (through J_rel_) that was translated by the Na^+^/Ca^2+^ exchanger (I_Naca_) into the depolarization of the membrane potential, thus determining the DAD and the anticipated AP.

We also identified models that, in spite their regular AP profiles when paced at CL = 1,000 ms, developed DADs with fast pacing at CL = 300 ms and one long beat at CL = 10,000 ms (Figure 7). Figures 7B,C show two illustrative models developing one DAD and one anticipated AP. In both models the mechanism is the same. Fast pacing induced Ca^2+^ accumulation in the SR (the sarcoplasmic Ca^2+^ concentration in steady state was 1.85 and 1.65 mM, respectively), causing a slow Ca^2+^ efflux from SR before the DAD/anticipated AP by means of the Ca^2+^ leakage flux from SR (J_leak_), that slowly increased [Ca^2+^]_i_ and the Ca^2+^ concentration in the subspace ([Ca^2+^]_SS_). Consequently, the RyR-sensitive channels sensed an increased [Ca^2+^]_SS_ and opened spontaneously (transition 0 to 1 of the RyR_o_ gating variable), allowing a Ca^2+^ efflux from SR via J_rel_. This Ca^2+^ release triggered a remarkable inward current by I_NaCa_ that depolarized the membrane potential, thus resulting in the DAD/anticipated AP.

**FIGURE 8:**
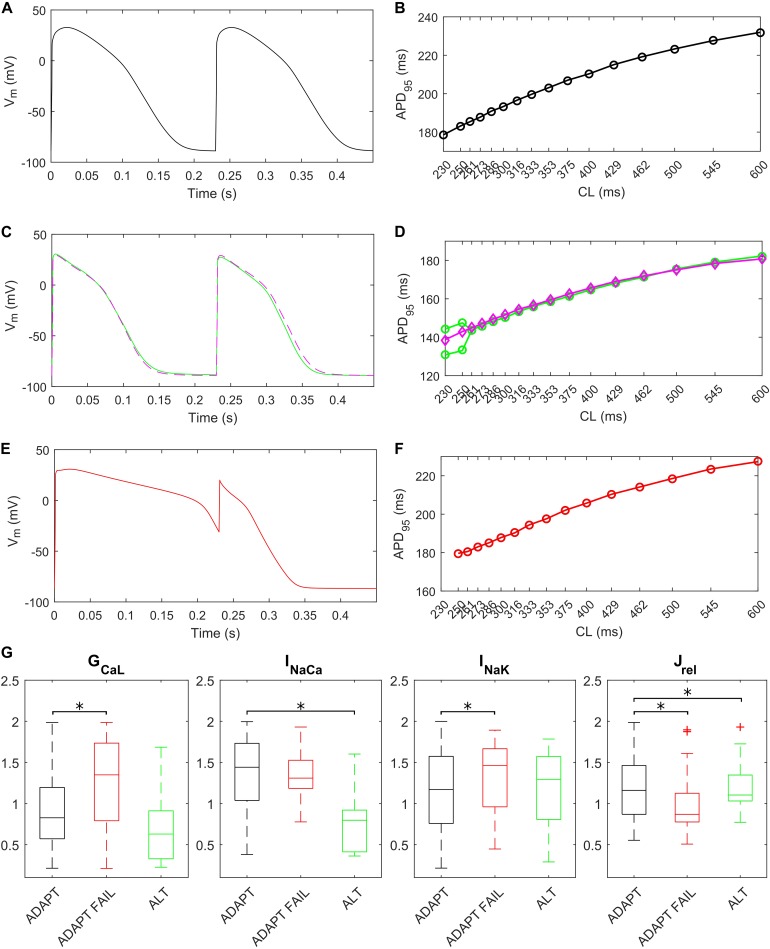
Repolarization abnormalities – alternans. Action potentials (APs) at different cycle lengths (CLs) (pacing at 30 s if CL ≥ 300 ms, otherwise 15 s) and APD_90_-CL relationship for three models from the *in silico* population. The model (black) in **(A,B)** belongs to the ADAPT class. The model (green) in **(C,D)** from the ALT class showed alternans and produced a bifurcation. **(E,F)** Show an ADAPT FAIL model (red, whose AP fails to adapt for CL shorter than 250 ms). The magenta trace in **(C,D)** show the alternans suppression due to 30% J_up_ upregulation. **(G)** Distribution of the scaling factors showing statistically significant differences (^∗^*p* < 0.05) between the three categories: models adapting to changes in pacing rate (ADAPT, black), models failing to adapt (ADAPT FAIL, red) and models developing alternans (ALT, green). Red crosses represent outliers.

The fast pacing rate protocol we used to trigger alternans resulted in 287 ADAPT models, 28 ADAPT FAIL models and 14 ALT models. 13 models were excluded from this analysis since their intracellular ion concentrations were out of the boundaries defined in the Materials and Methods section. Figure 8 shows three illustrative models for the ADAPT, ADAPT FAIL, and ALT groups. In Figures 8A,B, a representative ADAPT model (the baseline BPS2020) shows a progressive shortening of APD_95_ in response to the pacing rate increment. Figure 8C,D show a representative ALT model that produces a bifurcation. Finally, Figures 8E,F show a representative ADAPT FAIL model whose AP fails to adapt for CLs shorter than 250 ms. In ADAPT FAIL models, the next stimulus is overlapped more and more to the previous AP. In the 14 models showing alternans, we observed that J_rel_ failed to recover from inactivation (Supplementary Figure S11), or that the pacing failed to trigger I_Na_ (and consequently I_CaL_, Supplementary Figure S12). In both cases we observed one Ca^2+^ transient every other AP. We also tested whether a J_up_ increment in the ALT models would have suppressed this phenomenon, since Cutler et al. (2009) showed that 30% upregulation of J_up_ suppressed alternans. In fact, following a J_up_ increment, 5 of the 14 ALT models stopped developing alternans, 1 failed to adapt, 5 still developed alternans at higher pacing rate and in 2 no differences were observed. One model was discarded due to out-of-range intracellular concentrations. Therefore, our populations of models confirmed the role of J_up_ in suppressing/reducing alternans, as also showed by O’Hara et al. (2011). Figures 8C,D (magenta line) illustrate the alternans protocol results obtained with 30% J_up_ upregulation. Figure 8G shows the distribution of the maximum conductances/currents that are significantly different from the ADAPT class (*p* < 0.05).

## Discussion

In this study we present the BPS2020 model, an updated version of the ORd human ventricular AP model (O’Hara et al., 2011): our primary aim was to develop a model able to simulate the physiological inverse APD-[Ca^2+^]_o_ dependence, observed both *in vitro* and *in vivo* (Severi et al., 2009), and to reproduce the *in vitro* experiments at the correct [K^+^]_o_. The ORd model is currently considered the *gold standard* among the human ventricular AP model, since it was created using experimental data from over 100 undiseased human hearts, and validated against a plethora of different protocols. However, this model does not reproduce the inverse APD-[Ca^2+^]_o_ dependence. Therefore, it cannot be used in those cases where the effect of [Ca^2+^]_o_ variation on the AP is under investigation. The BPS2020 model fills this gap, fixing this mechanism without losing the ability to simulate the *in vitro* experiments reproduced by the original ORd model (e.g., APD rate dependence, restitution, response to administration of drugs). The major changes in BPS2020 compared to ORd are: (i) a novel Markov formulation for I_CaL_; (ii) a single compartment description of SR; (iii) a modified J_rel_ formulation, as in Paci et al. (2018b); (iv) the dynamic I_Kr_ formulation from Li et al. (2017); (v) the optimization of the maximum conductance of the other currents to better fit the *in vitro* data from O’Hara et al. (2011), both in control conditions and under drug block.

### APD Changes Induced by Extracellular Ca^2+^ Variations and Implications on Intracellular Ca^2+^ Handling

The APD dependence on [Ca^2+^]_o_ variations should be considered in all clinical contexts where electrolyte modifications occur, since APD changes are very important triggers for arrhythmia onsets. Unfortunately, most of the published human AP models do not take this dependence into account, and therefore respond in a non-physiological way: [Ca^2+^]_o_ increases lengthen APD, and vice versa.

**FIGURE 9:**
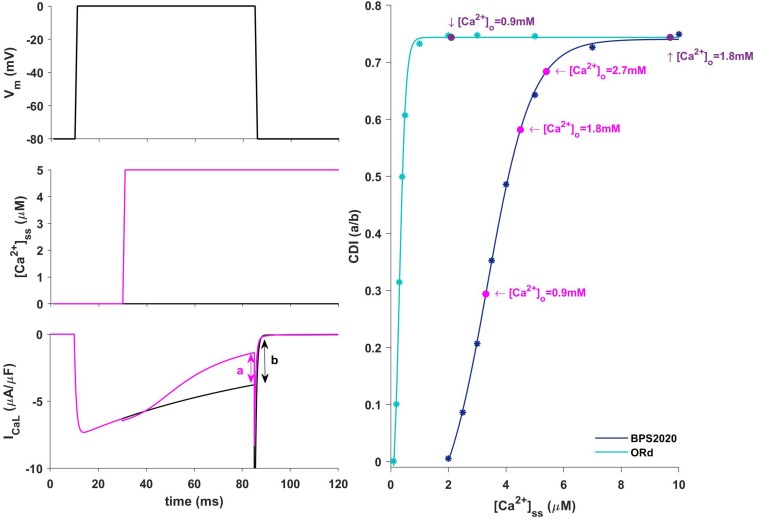
Ca^2+^-dependence inactivation. Left: framework for isolating the Ca^2+^-dependent inactivation (CDI) (from Limpitikul et al., 2018). Top: voltage step. Middle: [Ca^2+^]_ss_ example of concentration step. Bottom: I_CaL_ current in response to voltage activation at baseline [Ca^2+^]_ss_ (black) and in response to a [Ca^2+^]_ss_ step (magenta). Right: Steady state CDI (evaluated as a/b) as a function of [Ca^2+^]_ss_ with ORd (light blue) and BPS2020 model (dark blue), fitted with a Boltzmann curve; CDI quantification are also highlighted for different [Ca^2+^]_o_ values (magenta for BPS2020 and purple for ORd).

In our BPS2020 model, the APD-[Ca^2+^]_o_ dependence has been corrected: the original L-type Ca^2+^ current has been replaced by a new Markov model, where the CDI sensitivity to intracellular Ca^2+^ has been strengthened. Indeed, we found that the APD-[Ca^2+^]_o_ inverse relation is mainly mediated by the sensitivity of CDI to [Ca^2+^]_i_, in turn modulated by [Ca^2+^]_o_ variations. Upon changes of [Ca^2+^]_o_ two counteracting effects are elicited, both affecting I_CaL_: an increase in the driving force, which would promote APD prolongation, and an increase in CDI, which would promote a faster I_CaL_ inactivation and APD shortening. The latter prevails, thus leading to the physiological inverse relation. By quantitatively analyzing CDI using the approach by Limpitikul et al. (2018) we found that: (i) the “CDI strength” (i.e., the maximum amount of current that can be inactivated by CDI) is exactly the same in the ORd and our models (74%); (ii) the CDI sensitivity, i.e., changes in CDI strength around the working point, is definitely different. In the ORd model, the working point is placed into the superior saturation part of the nonlinear CDI curve, making the local sensitivity (slope) almost null. On the contrary, in BPS2020, the local sensitivity curve remains steep for the entire intracellular Ca^2+^ range. These results are shown in Figure 9. Sigmoidal models are quite common to represent physiological mechanisms, and the working point is usually in the high-gain range, rather than in the saturation range. Our results suggest that a poor local sensitivity of the CDI inactivation mechanism could be the main reason why most cardiac models, whose domain of applicability is limited to fixed standard [Ca^2+^]_o_ = 1.8 mM, fail to reproduce the physiological APD-[Ca^2+^]_o_ dependence.

We also tested if some other mechanisms influence the APD-[Ca^2+^]_o_ dependence, following the same approach used by Grandi et al. (2009), i.e., by removing the Ca^2+^ dependence from the I_Ks_ formulation, and varying the I_NaCa_ amplitude (−30% and +100% of its nominal value). In both cases, the correct APD-[Ca^2+^]_o_ dependence was maintained (see Supplementary Figure S15, left and right panel, respectively), therefore excluding both I_Ks_ and I_NaCa_ as relevant factors in modulating APD vs. [Ca^2+^]_o_.

By itself, the increased CDI sensitivity in BPS2020 had a negative impact on the restitution curve (S1S2 protocol). This was caused by the relative slow Ca^2+^ diffusion between the two SR compartments (time constant = 100 ms in ORd). When considering short diastolic intervals in the restitution protocol, the junctional SR doesn’t have time to refill, thus leading to a very small Ca^2+^ release. This has not a big effect in ORd, while in BPS2020 – where the sensitivity of CDI to changes in intracellular Ca^2+^ is increased – a smaller Ca^2+^ release leads to a significant decrease of CDI, with a consequent, unphysiological, AP prolongation at short diastolic intervals. A very fast diffusion (as described by a single SR compartment) was sufficient to restore the physiological behavior at all the pacing rates, and this is why in BPS2020 we decided to use a single SR compartment. To enforce this observation we tested the role of Ca^2+^ diffusion within the SR by restoring a SR two-compartments (JNS+NSR) in BPS2020, with the original diffusion time constant used in ORd (100 ms), and with faster diffusion (10 and 1 ms). As shown in Supplementary Figure S9, the physiological restitution curve is not reproduced when considering 100 ms (dashed line), but it is progressively restored when considering smaller time constants (dashed-dotted and solid line, for 10 and 1 ms, respectively). This result supports the new single compartment SR as a reasonable approximation of the diffusion process taking place within the complex SR structure. Indeed, a similar description is used also by other human ventricular models (Grandi et al., 2010; Paci et al., 2017).

At the same time, the assumption of two SR compartments is not biologically justified, since the SR is not anatomically divided into two substructures, and the experimental and modeling results still contain some controversy on the velocity of free Ca^2+^ diffusion within the SR (Bers and Shannon, 2013), with larger diffusion coefficient (quite consistent with a single compartment description) describing better the available data. No detectable [Ca^2+^]_SR_ gradients between SR release sites and Ca^2+^ uptake sites during normal global Ca^2+^ release have been reported by Bers’ Lab (Picht et al., 2011). Furthermore, potential differences in species have yet to be examined in full. Our simulation results fully support the idea of very rapid availability of SR Ca^2+^ for global release in normal conditions.

In the ORd model, the SR Ca^2+^ release flux J_rel_ via ryanodine receptor is proportional to the I_CaL_ current. With such direct dependence of J_rel_ on I_CaL_ excitation-contraction coupling models are not able to produce pro-arrhythmic triggers, such as DADs (Fink et al., 2011). For this reason we decided to replace the J_rel_ equations with a phenomenological formulation (Koivumäki et al., 2011; Paci et al., 2018b). The BPS2020 model is now capable of reproducing complex features of RyR Ca^2+^ release, i.e., the dependence of the release on both [Ca^2+^]_i_ and [Ca^2+^]_SR_ and the adaptation of the RyR open probability dependence on [Ca^2+^]_i_ (Koivumäki et al., 2011). Since the quite unnatural direct dependence of J_rel_ on I_CaL_ is usually assumed to confer graded release on phenomenological models of CICR, we tested if it was still possible to reproduce graded Ca^2+^ release with our new formulation of J_rel_. This is actually the case for voltages below 0 mV while the model strays from experimental results for positive voltages (see Supplementary Figure S7): this feature of the model deserves further quantitative investigation in the future.

### Comparison Against the Experimental Data Used to Validate the ORd Model

We challenged our model by comparing our simulations with all the experimental data that the ORd model simulated in O’Hara et al. (2011). This includes a large variety of protocols: AP morphology in physiological conditions, APD rate dependence and restitution, both in control conditions and with specific channel blockers, transmural heterogeneity, APD accommodation, [Ca^2+^]_i_ and [Na^+^]_i_ rate dependence, EADs induced by I_Kr_ block during slow pacing, and AP and Ca^2+^ alternans induced by high pacing rate.

The BPS2020 model reproduced all these data, with an accuracy at least as good as the ORd model, and – in most cases – better, as shown by comparing the root mean square error to quantify the distance between experimental data and simulations (see Supplementary Table S2).

It is worth noting that many *in vitro* experiments were actually reproduced significantly better by BPS2020 than ORd, and that our simulations with the ORd model differ from those presented by O’Hara et al. (2011). This is mainly due to an accurate revision of the electrolyte concentrations used in the different simulated conditions. In fact, to achieve a proper comparison between experiments and simulations, it is crucial to simulate the cardiac AP by imposing the same extracellular concentrations used in the corresponding experimental protocol. However, this constraint has often been neglected in the past. All the comparison between experiments and simulations presented here followed this principle. In O’Hara et al. (2011), comparisons with experiments were instead simulated with basal extracellular concentrations ([Na^+^]_o_ = 140 mM, [Ca^2+^]_o_ = 1.8 mM and [K^+^]_o_ = 5.4 mM), although the *in vitro* data were mainly recorded with [KCl] = 4 mM in the bath solution.

### Comparison Against Other Human Models

Although several human ventricle AP models were developed and published in the past two decades, the ORd model is currently considered as the state-of-the-art in the field, based on its extensive validation with human data. Therefore, we have performed a stringent comparison between the two models, while we considered a systematic benchmarking with all the available models beyond the scope of this paper.

Two recent studies presented optimized versions of the ORd model: the first to fit various LQTS profiles (Mann et al., 2016), the second to optimize the ORd model for its use in safety pharmacology, within the CiPA initiative (Dutta et al., 2017a) (ORd CiPA). The scaling factors they proposed for the maximum conductances of the main ionic currents are different from the ones in our model: I_Ks_ is increased by 5.75 in Mann et al. model (Mann et al., 2016) and by 1.87 in Dutta et al. (2017a). Ours is increased by an intermediate amount (by 2). I_Kr_ is increased in the BPS2020 model as it is in Dutta et al. (2017a), but the increase in the former is slightly greater. I_NaL_ is unchanged in the Mann model while it is scaled by 2.661 in the Dutta model; in line with the latter (O’Hara et al., 2011), we incremented its conductance by 2.8. In the present model, I_NaCa_ and I_NaK_ were increased (by factors of 2.4 and 2, respectively) as done by Mann et al. (2.95 and 9.12) (Mann et al., 2016). In addition, I_K1_ was decreased in the BPS2020 model.

APD rate dependence and APD-[Ca^2+^]_o_ relationship for ORd, BPS2020, ORd CiPA (Dutta et al., 2017a) and Grandi et al. (2010) models are compared in Supplementary Figure S18. As shown, the ORd CiPA model is very similar to the ORd one, and both ORd CiPA and Grandi cannot reproduce the physiological APD-[Ca^2+^]_o_ relationship. In addition, Supplementary Figure S17 shows a comparison of the AP and the major ionic currents for BPS2020, ORd, and ORd CiPA; simulations were in single ENDO cells paced to steady state at CL = 1,000 ms.

Another modification of the ORd model has been very recently published by Tomek et al. (2019). The main aim driving the development of that model (simulation of the negative inotropic effect of sodium current block) was different from the one of BPS2020 (APD dependence on [Ca^2+^]_o_). As a matter of fact, both models have been used to investigate and integrate into the equations additional knowledge of basic cardiac cell electrophysiology (some of them in the form of model-generated hypotheses), e.g., sensitivity of CDI to intracellular [Ca^2+^], extraction of the activation curve from I–V data, to mention only a couple. However, a rigorous comparison of the two models, as well as the possibility of combining them into a comprehensive AP model, is far beyond the scope of the present work.

### Simulation of the Biological Variability of Human Cardiac AP Through a Population of Models

The population of models approach (Britton et al., 2013; Muszkiewicz et al., 2015) has been used for two different purposes: (i) to explore the ability of the model to reproduce the variability observed in human experimental data in control conditions; (ii) to assess the variability in drug response and occurrence of pro-arrhythmic events, following I_Kr_ block. The first goal was achieved by first varying the maximum conductances of the main ionic currents around their nominal values (Figure 5A), under the assumption that the variability mostly depends on the difference in the ionic channel expressions (Groenendaal et al., 2015). Then, model outputs were calibrated against experimental data in order to select models with AP biomarkers in the physiological range, and discard the others. In this way, each model becomes a representation of a viable cell within the plausible boundaries of the biological variability observed in the population (Britton et al., 2013; Passini et al., 2015). Of note, the population generated from the BPS2020 model by varying maximum conductances in the (20, 200%) range, covers the range of experimentally measured biomarkers, especially those related to the repolarization phase (APD at different level of repolarization and triangulation, Figure 5C). This suggests that (20, 200%) is a good variability range to develop a population of *in silico* adult cells. This directly links to the second goal. We used our population to assess the capability of the BPS2020 to reproduce phenomena whose occurrence cannot be quantified in terms of mean value (e.g., EADs, DADs, and alternans), and that are not easy to reproduce with the average undiseased cell model. In vivo, EADs, DADs, and alternans are elicited by specific ‘stressfull’ protocols (using drugs, very low or very high pacing rates, etc.). But even in these challenging conditions only few cells show them (for example, as shown in Guo et al., 2011; Coppini et al., 2013). This, of course makes sense, since healthy cells have to be very robust with respect to pro-arrhythmic phenomena occurrence. In the same way, a computational model aiming to reproduce a healthy cardiomyocyte should not necessarily reproduce these arrhythmias and the population of models approach is particularly well suited for these investigations The fact, with our *in silico* population we were able to obtain three different pro-arrhythmic abnormalities (EADs, DADs, and alternans), providing further validation of the BPS2020 model.

### Limitations

The BPS2020 model was extensively validated based on the experimental protocols shown in the original O’Hara et al. paper (O’Hara et al., 2011). However, some limitations can be highlighted, and they should be investigated in more in detail in the future. In the ORd model the I_CaL_ I–V curve does not perfectly fit the positive potentials and this remains the case for the BPS2020: this shortcoming could lead to I_CaL_ overestimation at positive potentials. Although the new J_rel_ formulation, with no direct dependence on I_CaL_, was still able to reproduce graded Ca^2+^ release in good agreement for negative voltages, for positive voltages the model strays from experimental data: a detailed investigation of this phenomenon, including a comparison with local control models of Ca^2+^ release, would be necessary but is beyond the scope of the present work. Another limitation is that in our formulation, the RyR opening dependence on Ca^2+^ is very steep (Supplementary Figure S8). Li and Chen (2001) showed that the RyR opening dependence on Ca^2+^ in lipid bilayers is indeed very steep for low Ca^2+^ concentrations, however, our steady-state RyR opening curve is actually even steeper than the experimental one.

The weakness of CDI at negative potentials is a limitation of our model that could be improved in the future. However its impact under AP is likely very limited, since the membrane potential crosses very quickly the voltage interval [−20, −10] mV, firstly during the upstroke and secondly during the late repolarization phase. Besides, the amount of elicited current at −10 mV would be very small (see I–V curve in Figure 1B). The CDI slowness coming from FRC results is another limitation: in the future it could be fixed, further enhancing its role in modulating APD in case of extracellular Ca^2+^ changes.

Due to the relatively poor availability of human cardiomyocytes data, it is reasonable to exploit all those available to the utmost, in order to obtain a model with properties as close as possible to human cells. For this reason, we used all the available human data for model calibration. However, the separation of model calibration and validation (on independent data sets) could be taken into account, once more human data will be available.

## Conclusion

The new BPS2020 model correctly simulates the inverse APD-[Ca^2+^]_o_ dependence, generally not considered by the human ventricular models available in literature, including ORd. BPS2020 accurately simulates a variety of *in vitro* experiments, and it also takes into consideration the actual electrolyte concentrations used in the experimental setup. These are the experiments used to develop and validate the ORd model, of which BPS2020 represents a modified version. Furthermore, the new Ca^2+^ release formulation enables BPS2020 to produce DADs. In conclusion, the BPS2020 model expands the domain of applicability of the current ORd model, by adding the possibility to explore changes in ventricular electrophysiology induced by electrolyte changes, e.g., effects of hemodialysis or pathological changes in Ca^2+^ concentrations. Therefore, the BPS2020 model can be used to simulate and investigate a variety of conditions, and constitutes what we propose to be deemed an advanced general-purpose model of human ventricular cardiac electrophysiology.

## Data Availability Statement

All datasets generated for this study are included in the article/Supplementary Material.

## Author Contributions

CB, EP, and SS conceived and designed the study. CB and EP developed and validated the *in silico* model. MP and JH enabled the access to parallel computing facilities, developed the population of models, and run the *in silico* experiments on the population. CB, EP, MP, and SS analyzed the *in silico* data, prepared the figures, and drafted the manuscript. All the authors interpreted the results and revised the manuscript.

## Conflict of Interest

The authors declare that the research was conducted in the absence of any commercial or financial relationships that could be construed as a potential conflict of interest.
