# Supplementary material for: Simulation of the Effects of Extracellular Calcium Changes Leads to a Novel Computational Model of Human Ventricular Action Potential With a Revised Calcium Handling
Source: Front Physiol. 2020 Apr 15;11:314. doi: 10.3389/fphys.2020.00314 (PMC7174690; doi:10.3389/fphys.2020.00314)
Supplement: Supplementary file 1 [file Presentation_1.pdf]

Supplementary Material

1. Supplementary Figures

1.1 APD vs $[Ca^{2+}]_o$ experimental data

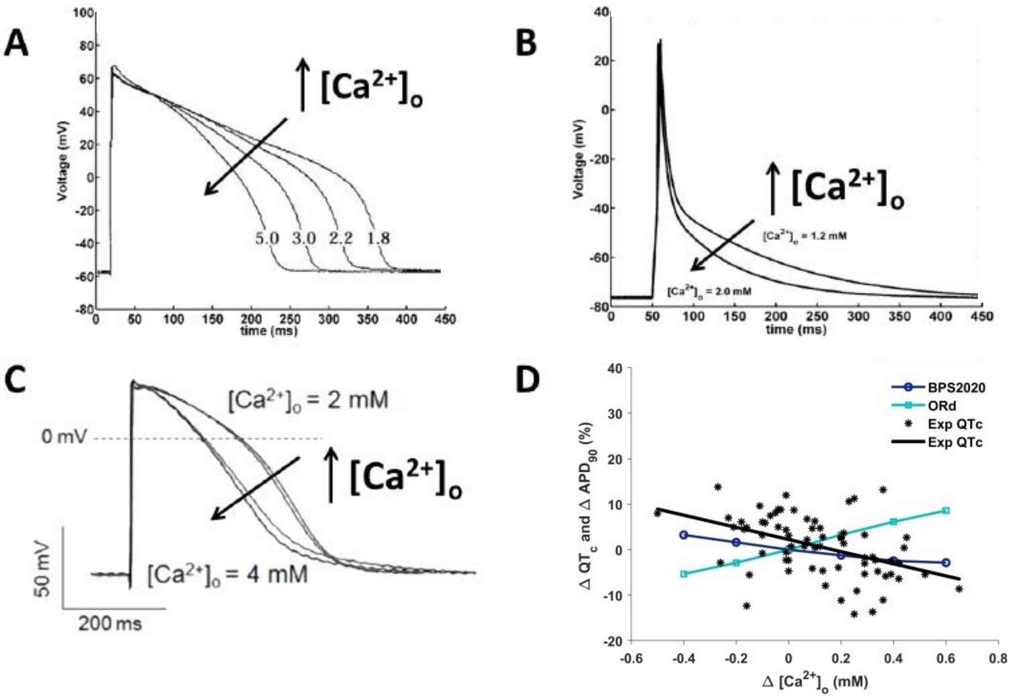

Supplementary Figure S1. Experimental Data from literature showing the inverse dependence of APD vs $[Ca^{2+}]_o$: (**A**) guinea pig ventricular cells (modified from (1)); (**B**) human atrial cells (modified from (2)); (**C**) human atrial cells (modified from (3)); (**D**) Comparison of $[Ca^{2+}]_o$ dependence of measured QTc interval and simulated APD. Scatter plot and regression line show the significant inverse correlation between QTc interval and serum $[Ca^{2+}]_o$ variations measured in different patients during haemodialysis therapy (data from (4)). Simulated APD values and polynomial interpolation were derived from data in Fig. 1B after normalization to the APD value obtained at the average pre-dialysis $[Ca^{2+}]$ (1.2 mM).

1.2 L-type Calcium Current (I_{CaL})

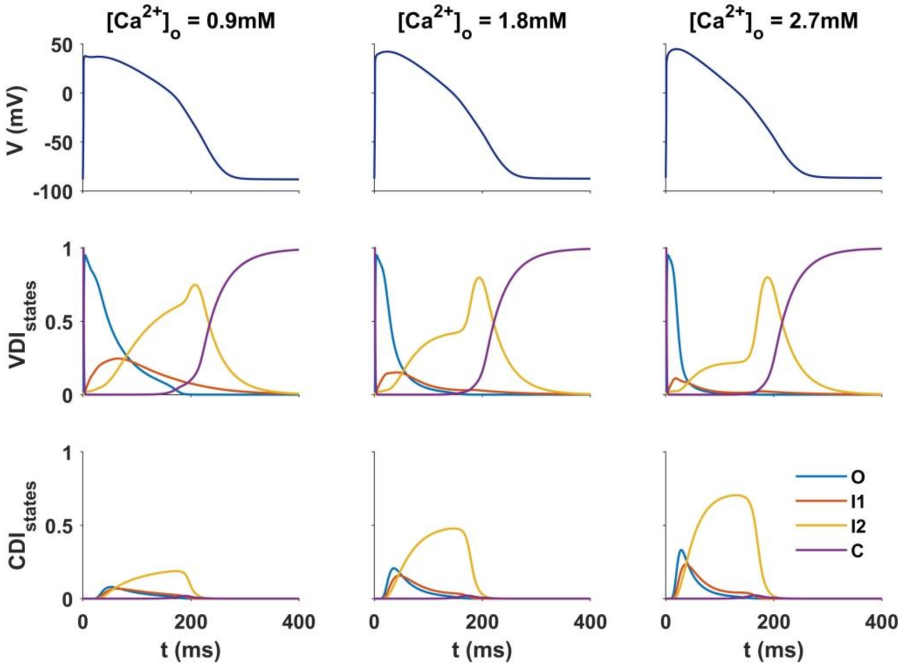

Supplementary Figure S2: I_{CaL} states occupancy versus different extracellular calcium concentration ($[Ca^{2+}]_o$=0.9mM left column, $[Ca^{2+}]_o$=1.8mM middle column and $[Ca^{2+}]_o$=2.7mM right column.

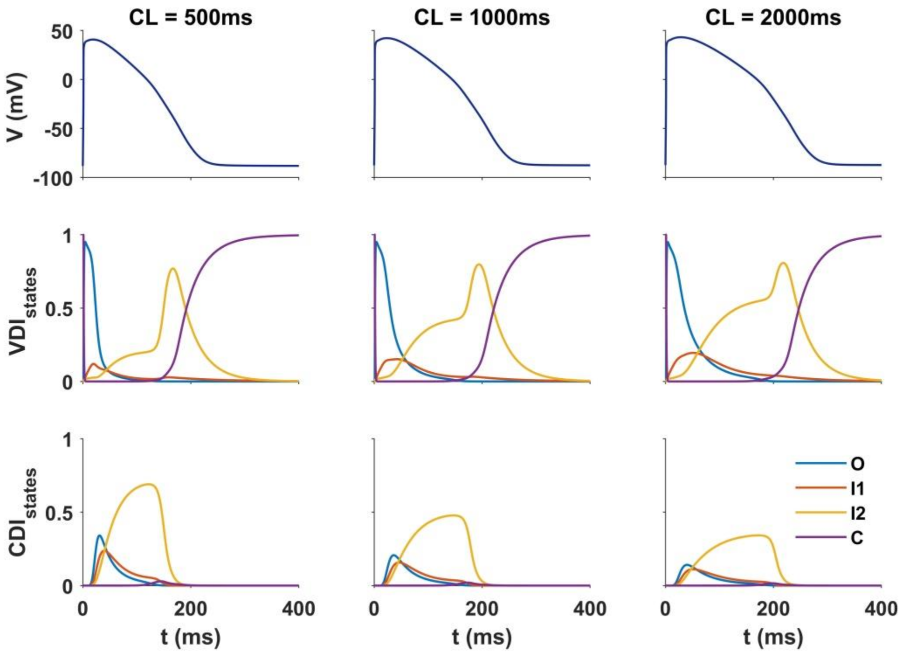

Supplementary Figure S3: I_{CaL} states occupancy versus different pacing frequencies (CL=500ms left column, CL=1000ms middle column and CL=2000ms right column.

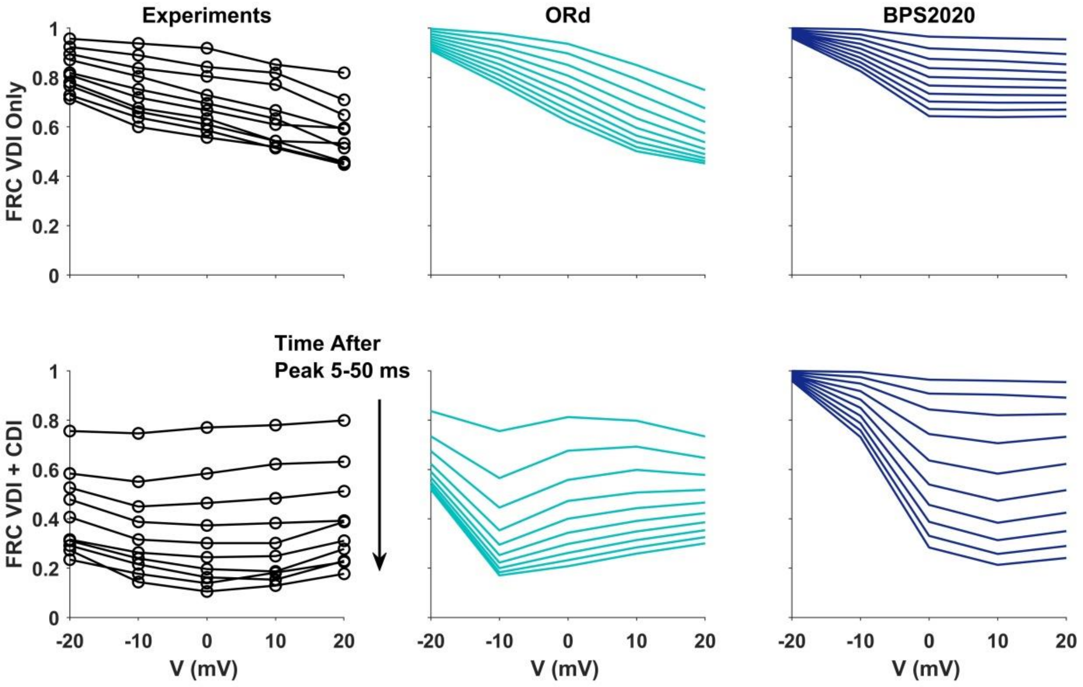

Supplementary Figure S4: Fractional remaining current. Experimental data on the left from (5); simulation with ORd model on middle panel, and simulation with BPS2020 on the right panel.

1.3 Ca^{2+} handling

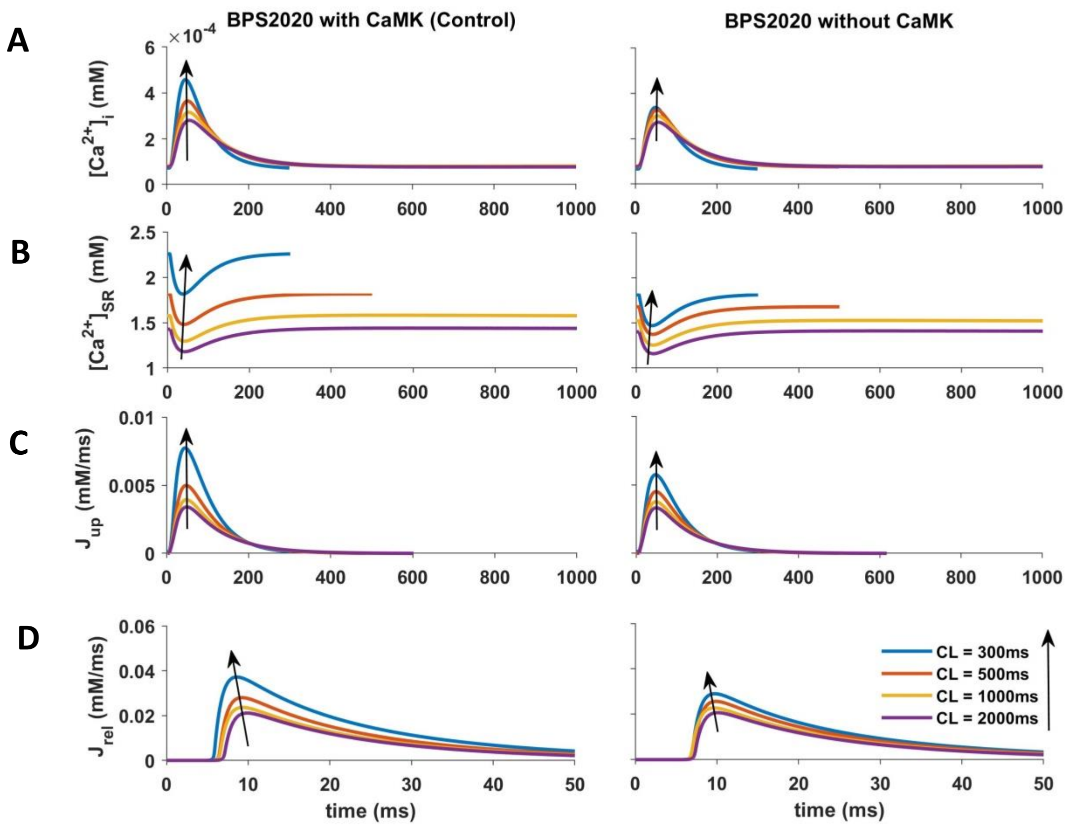

Supplementary Figure S5. Ca^{2+} cycling under control conditions (left) and without CaMK (right). CL changes are indicated by arrows. (**A**) $[Ca^{2+}]_i$. (**B**) $[Ca^{2+}]_{SR}$. (**C**) SERCA pump (Jup) and (**D**) Jrel.

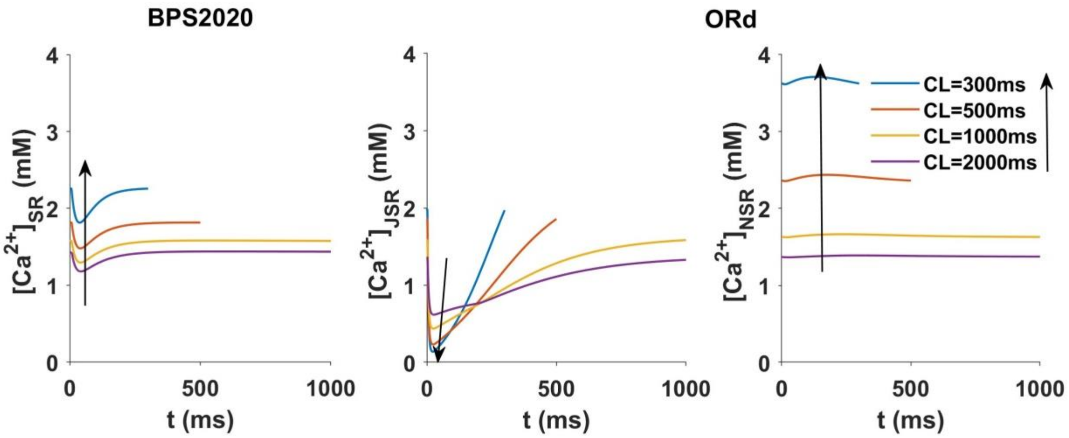

Supplementary Figure S6. Ca^{2+} load in the sarcoplasmic reticulum with BPS2020 (unique compartment, right panel), and with ORd model (junctional, middle panel; network right panel) in rate dependence (CL changes are indicated by arrows).

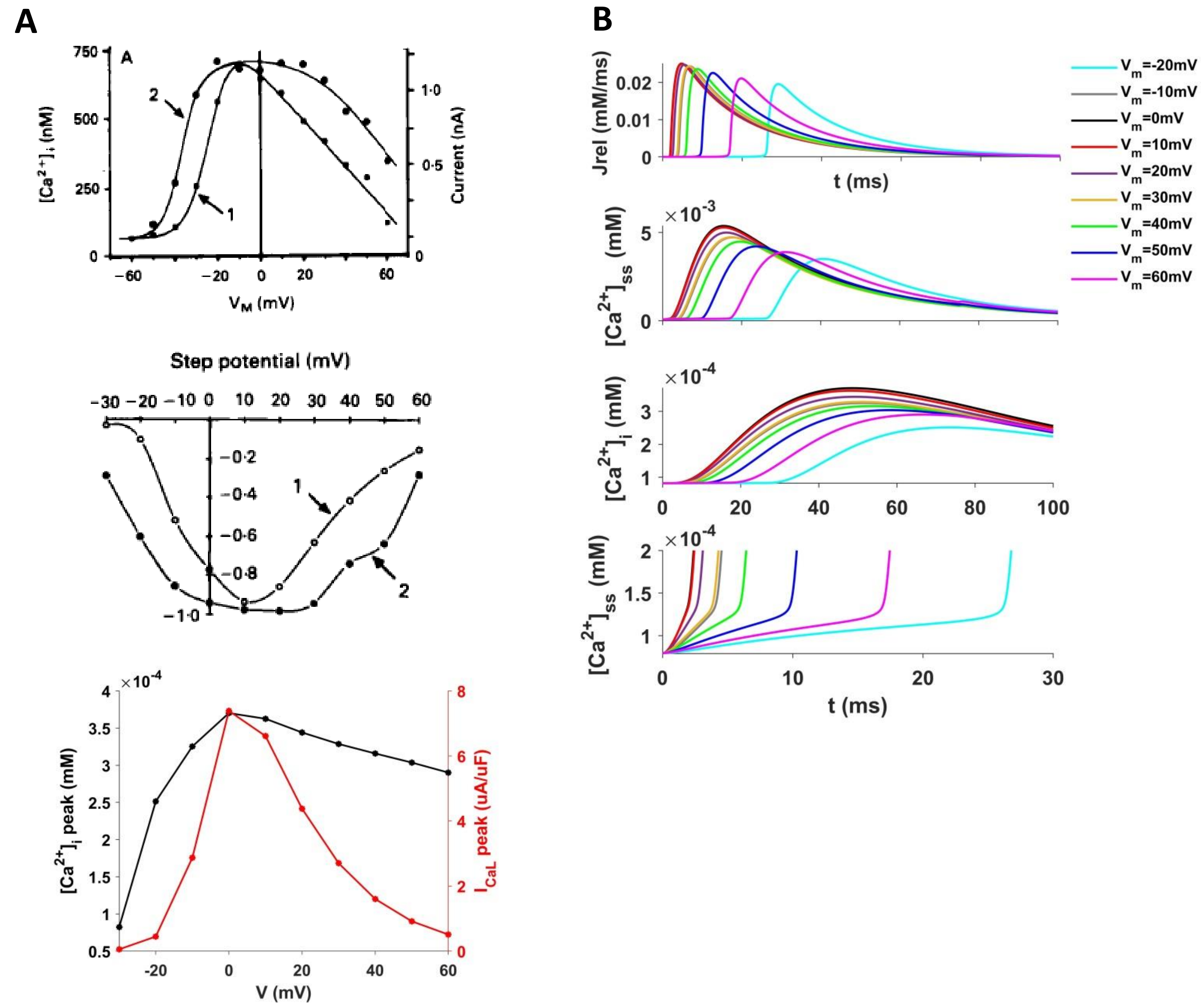

Supplementary Figure S7. (**A**) Top: voltage dependence of peak Ca^{2+} current (filled squares - curve 1) and peak Ca^{2+} transient (filled circles - curve 2) in rat ventricular myocytes. Middle: voltage dependence of peak Ca^{2+} current (open circles - curve 1) and peak Ca^{2+} transient (closed circles - curve 2) in feline ventricular myocytes (adapted from (6)). Bottom: voltage dependence of peak Ca^{2+} current (red) and peak Ca^{2+} transient (black) with BPS2020 model. (**B**) Top: Release vs time at different step voltages. Middle: $[Ca^{2+}]_{ss}$ and $[Ca^{2+}]_i$ peak transient vs time at different step voltages. Bottom: zoom of $[Ca^{2+}]_{ss}$ peak transient at different step voltages.

There is good agreement with experiments for negative voltages but the model strays from experimental results for positive ones. It can be observed that in the model: i) the amplitude of $[Ca^{2+}]_i$ peak seems to be inversely correlated to the delay in the calcium transient (CaT) upstroke; ii) the CaT

upstroke is obviously triggered by the calcium release from SR; iii) in turn, the release is triggered, through the CICR mechanism, by the increase in $[Ca^{2+}]_{ss}$; iv) the slow early increase in $[Ca^{2+}]_{ss}$ is driven by I_{CaL}, however a nonlinear relation seems to link I_{CaL} peak (bottom panel A) to the time-to-threshold of Cass (see zoomed bottom panel B), so that for positive voltages in spite of a large decrease in the I_{CaL} peak only a minor delay of the CaT is observed, and even at 60 mV the delay in CaT upstroke is significantly shorter than at -20 mV.

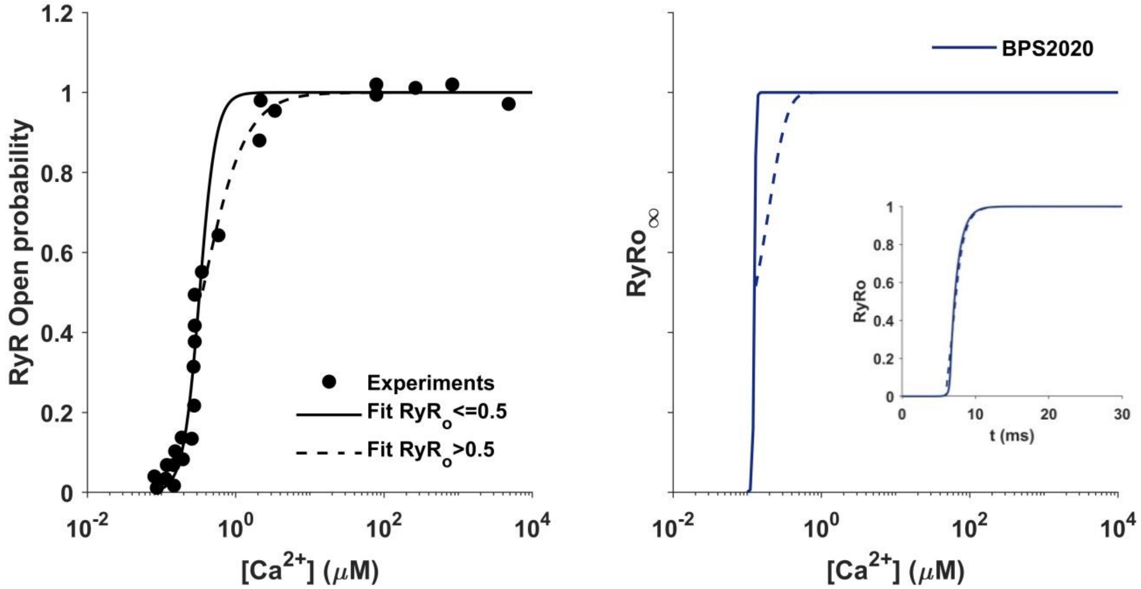

Supplementary Figure S8: RyR open probability vs $[Ca^{2+}]$ concentrations curve. Left panel: black circles experimental data from (7), black solid line fitting for RyR open probability <= 0.5 and dashed line fitting for RyR open probability > 0.5. Right panel: BPS2020 simulations. (blue line model control; dashed line simulated with a RyRo slope reduction (from 0.003 to 0.008) in the high part of the curve. Inset panel: RyR opening vs time.

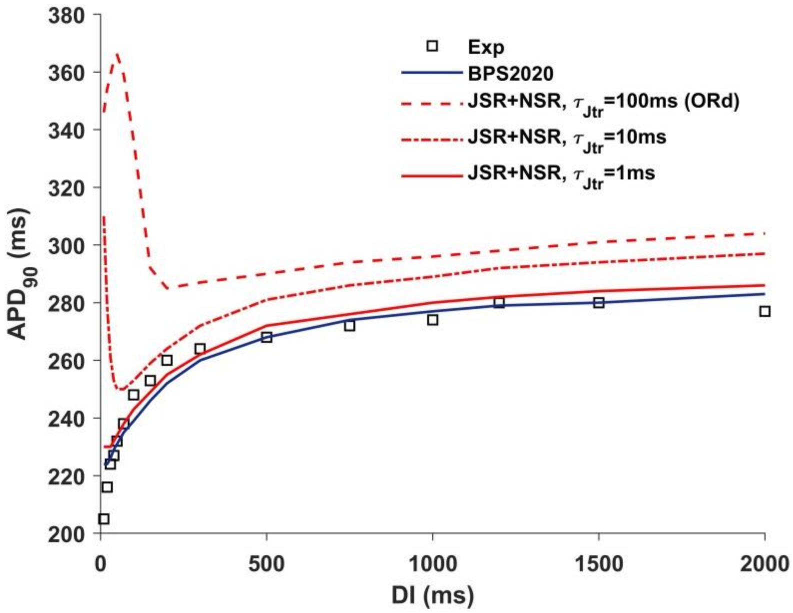

Supplementary Figure S9: APD restitution obtained with the S_1S_2 protocol (DI – diastolic interval). The red traces consider the two compartments formulation for the sarcoplasmic reticulum and the τ of diffusion between network and junctional has been modified: 100ms as ORd red dashed trace, 10ms red dashdotted and 1ms solid line. The BPS2020 model are blue line; experimental data from (5) are shown as black squares.

1.4 Transmural heterogeneity

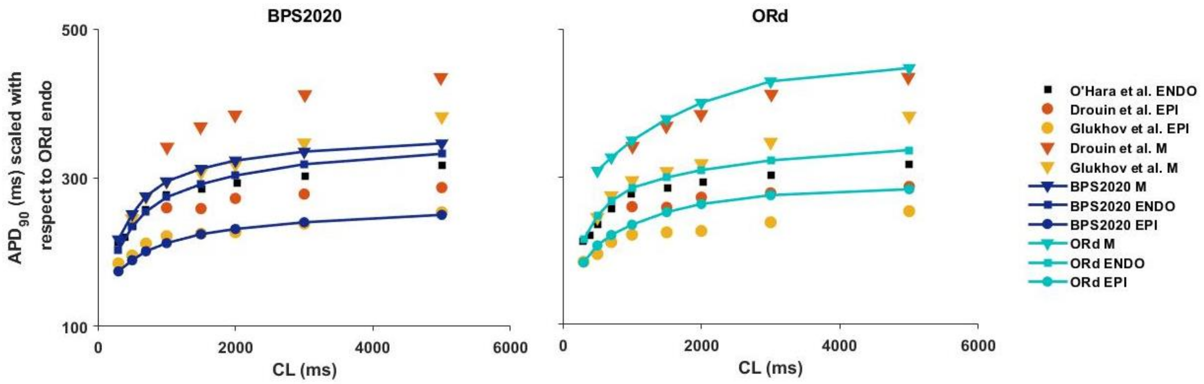

Supplementary Figure S10. Rate dependence of transmural cell type models in endocardial (squares), M (triangles) and epicardial (circles) myocytes with BPS2020 model (left panel) and ORd model (right panel). The red markers are from Drouin et al. (8) and the orange from Glukhov et al. (9) obtained as described by O'Hara (5), by scaling the endocardial data (white squares) by M/endo ratios. The black triangles (APD80) are derived with epi/endo and M/endo scaling. Light blue traces were simulated with the ORd model, dark blue traces with the BPS2020 model.

1.5 Alternans mechanisms

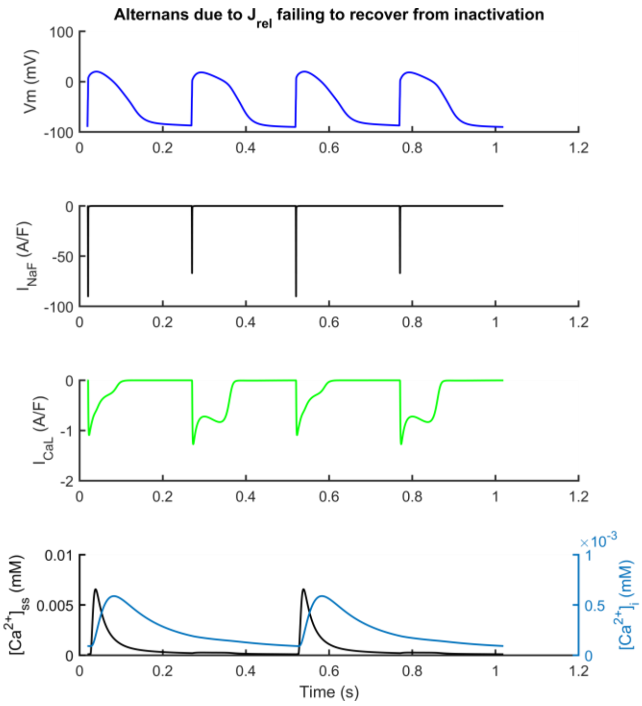

Supplementary Figure S11. Alternans due to failed recovery from inactivation of the Ca^{2+} release from Sarcoplasmic Reticulum (J_{rel}). Every second beat, the fast Na^{+} current (I_{NaF}) triggers an action potential, but J_{rel} is still inactivated from the previous beat (gate $RyR_c = 0$).

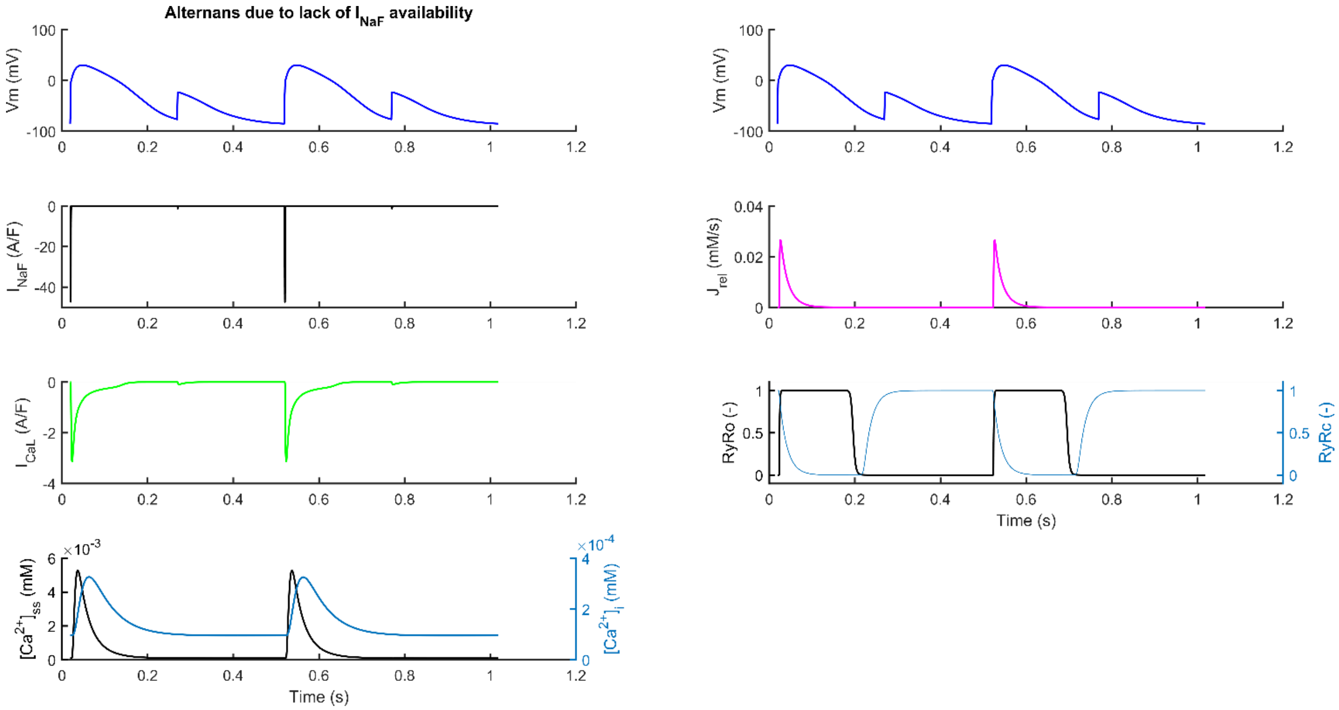

Supplementary Figure S12. Alternans due to lack of fast Na^+ current (I_{NaF}) availability. Every second beat, I_{Na} fails to trigger a full action potential. The Ca^{2+} release from Sarcoplasmic Reticulum (J_{rel}) has recovered from the previous beat, but the L-type Ca^{2+} current (I_{CaL}) is not triggered and the calcium-induced calcium release does not happen.

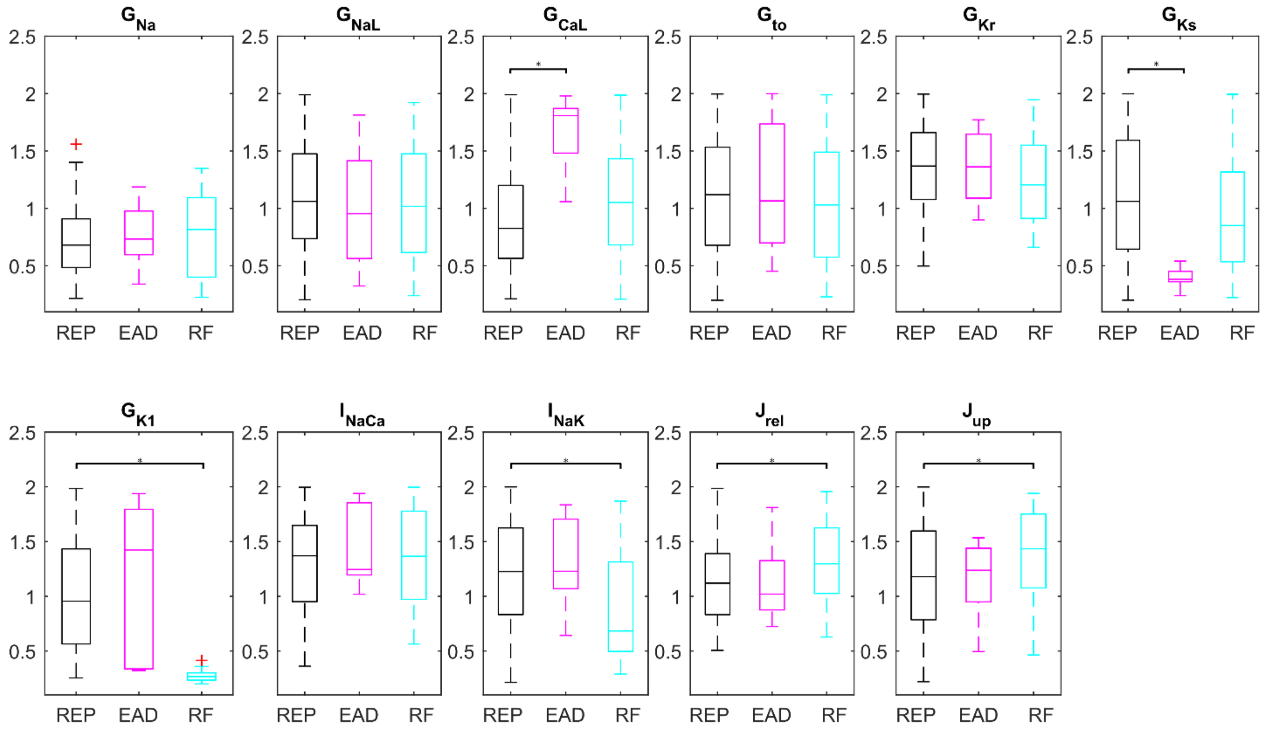

Supplementary Figure S13: Distribution of the scaling factors of the 11 sampled model parameters in the three groups of models showing different responses to dofetilide (0.1 μM, CL = 4,000 ms): repolarizing models (black, REP); models developing early afterdepolarizations (magenta, EAD) and models failing to repolarize (cyan, RF). Red crosses represent outliers.

1.6 AP dependence on extracellular concentrations

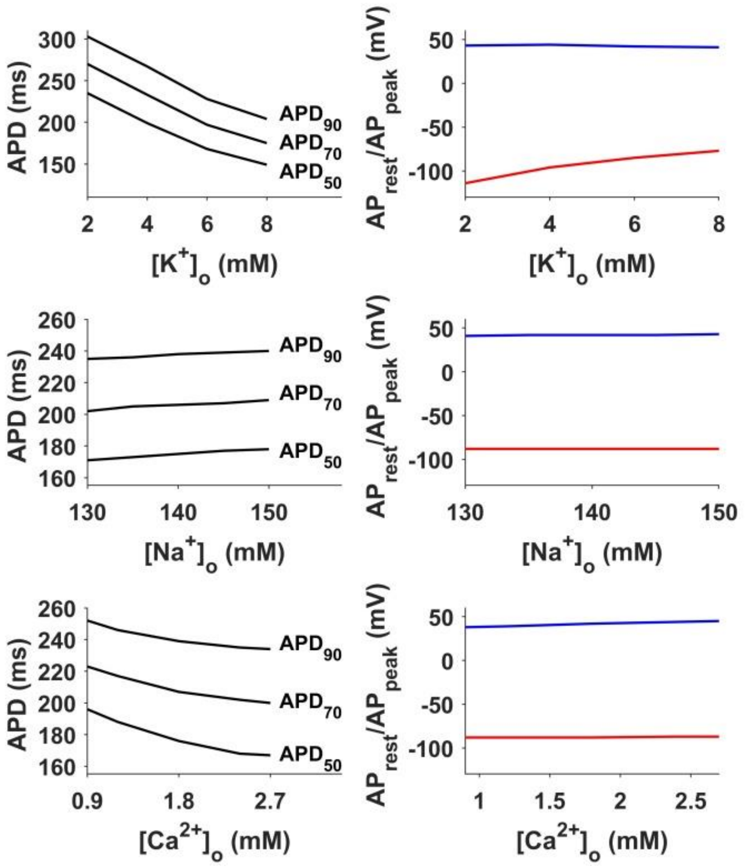

Supplementary Figure S14: AP biomarkers ($APD_{90,70,50}$ left panels; AP resting (red) and peak (blue) right panels) dependence on extracellular electrolytes. Upper panels: $[K^+]_o$; Middle panels: $[Na^+]_o$; Bottom panels: $[Ca^{2+}]_o$.

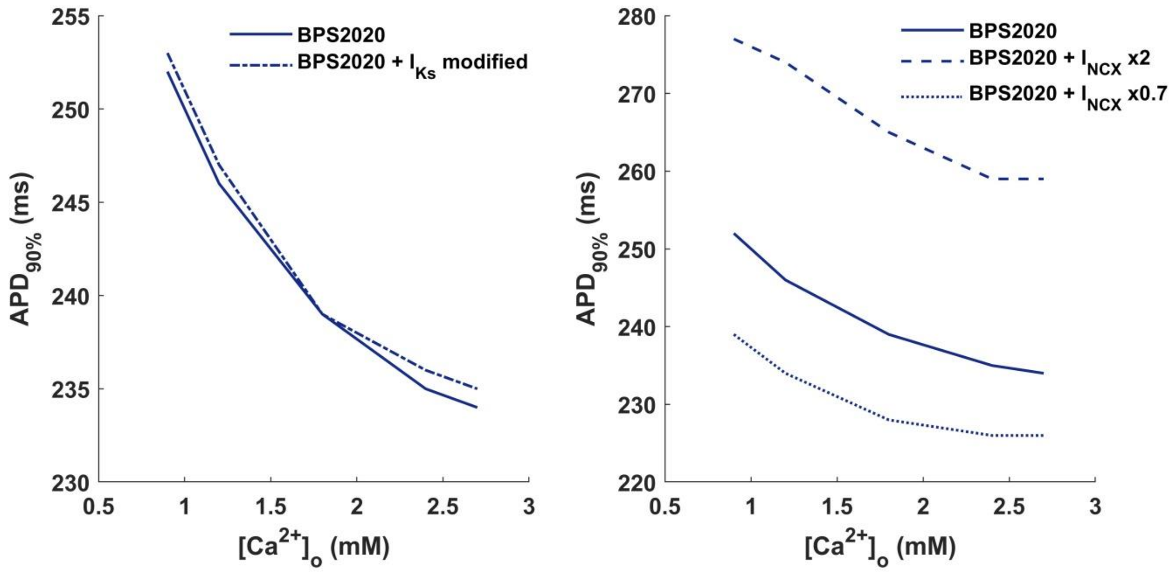

Supplementary Figure S15: Left panel: I_{Ks} effects on APD-$[Ca^{2+}]_o$ dependence (blue line BPS2020 model, dashed line BPS2020 by fixing the $[Ca^{2+}]_i$ to its diastolic value on I_{Ks} formulation). Right panel: Model sensitivity to I_{NCX} amplitude (blue line BPS2020 model, dashed line BPS2020 with a double increment of I_{NCX} and dotted line BPS2020 with a I_{NCX} reduction of 30%.

1.7 Effect of I_{NaK} on APD rate dependence

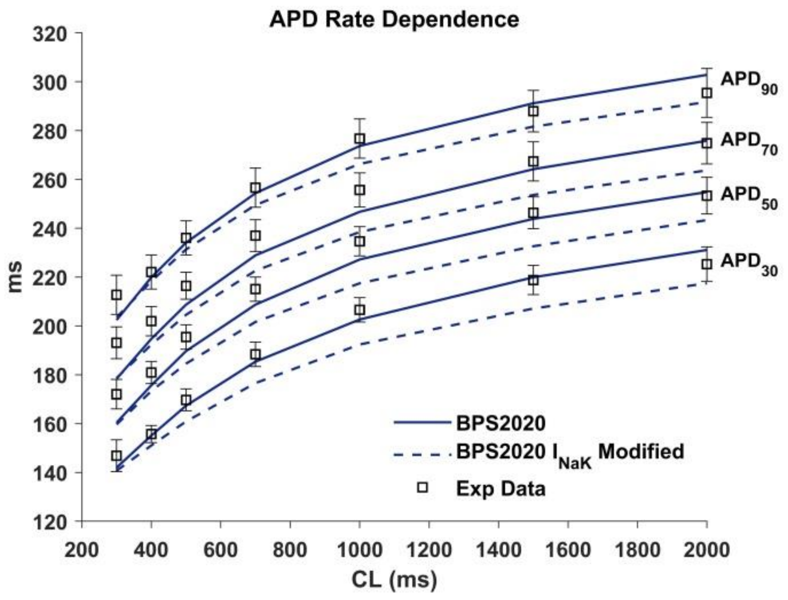

Supplementary Figure S16: Steady state APD rate dependence with BPS2020 model (blue line) and with BPS2020 restoring the I_{NaK} conductance as in (5) (dashed line). Experimental data form (5) are black squares.

1.8 Comparison against other human models

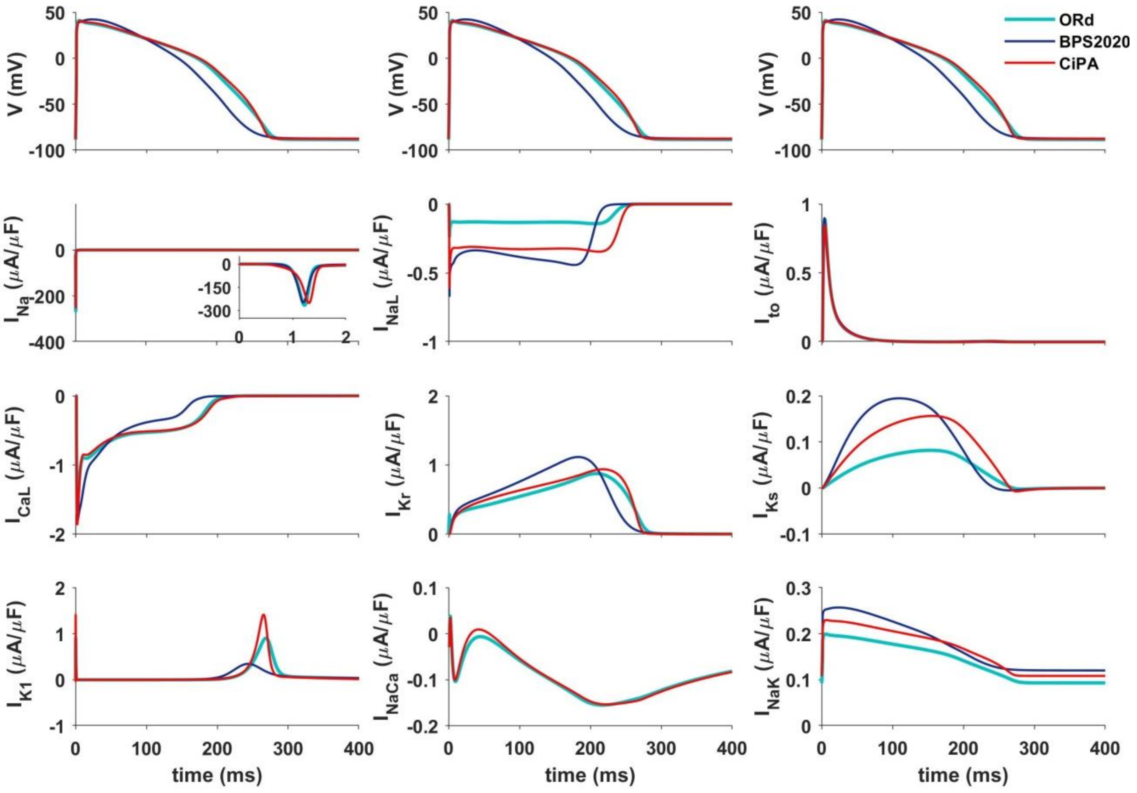

Supplementary Figure S17. Comparison with other human ventricular AP models. Single endo cell simulations from ORd (5), BPS2020, and ORd CiPA (10) models are light blue, dark blue and red lines, respectively. From left to right, top to bottom: AP, I_{Na} (inset shows peaks), late I_{Na}, I_{to}, I_{CaL}, I_{Kr}, I_{Ks}, I_{K1}, I_{NaCa}, I_{NaK}.

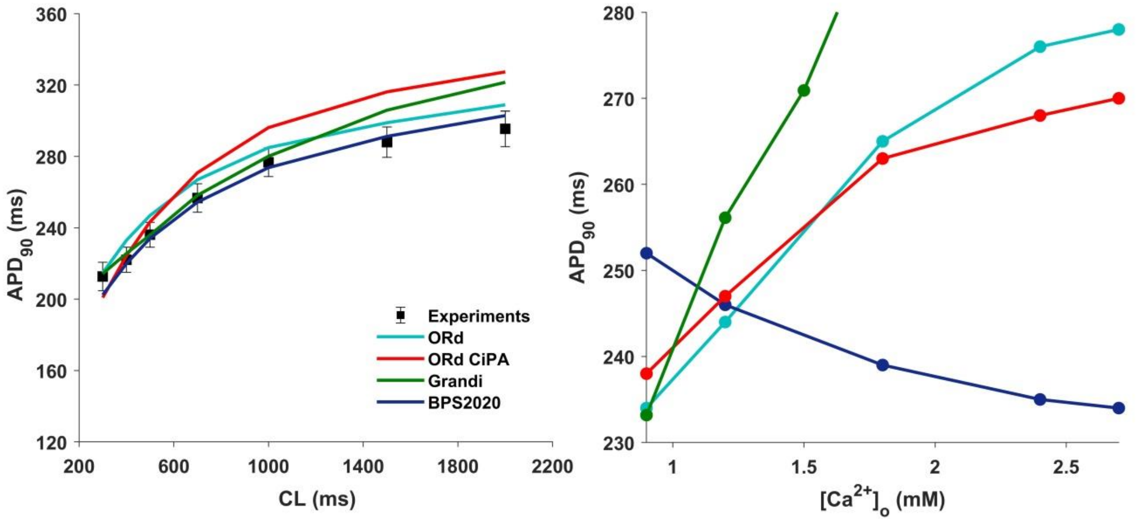

Supplementary Figure S18. Left: APD rate dependence comparison with other human ventricular AP models. Right: APD vs $[Ca^{2+}]_o$ relationship in ORd (light blue, (5)), BPS2020 (dark blue), ORd CiPA (red, (10)) and Grandi model (green, (11)).

2 Supplementary Tables

2.1 BPS2020 Human Model Transmural Heterogeneity

Table S1. Scaling factors for implementing the transmural heterogeneity. The bold indicated a value modified respect to ORd (5).

	Epi/endo	M/endo
G_{NaL}	**0.7**	1
G_{to}	4	4
P_{Ca}, P_{CaNa}, P_{CaK}	**1.4**	**2**
G_{Kr}	**1.1**	0.8
G_{Ks}	1.4	1
G_{K1}	1.2	1.3
G_{NaCai},G_{NaCass}	**1.2**	1.4
G_{NaK}	0.9	0.7
G_{Kb}	0.6	1
$J_{rel,NP}$, $J_{rel,CaMK}$	1	1.7
$J_{up,NP}$, $J_{up,CaMK}$	1.3	1
$\overline{[CMDN]}$	**1.2**	1

2.2 Quantitative comparison of the quality of fitting

Table S2. Root mean square error to quantify the distance between experimental data and simulations with the ORd and the BPS2020 models.

		Root Mean Square Error	
		ORd model	BPS2020 model
**Figure 3A**	APD rate dependence (ms)	13.5	6.3
**Figure 3A**	APD restitution (ms)	15.5	14.6
**Figure 3B**	I_{Kr} Block rate dependence (ms)	69.4	52.7
**Figure 3B**	I_{Ks} Block rate dependence (ms)	36.6	33.7
**Figure 3B**	I_{K1} Block rate dependence (ms)	16.4	35.5
**Figure 3B**	I_{CaL} Block rate dependence (ms)	16.8	17.3
**Figure 3B**	I_{NaL} Block rate dependence (ms)	42.0	30.0
**Figure 3C**	I_{Kr} Block restitution (ms)	52.3	11.3
**Figure 3C**	I_{Ks} Block restitution (ms)	16.2	16.0
**Figure 3D**	Normalized $[Na^+]_i$ vs freq (%)	11.0	3.0
**Figure 3D**	Normalized $[Ca^{2+}]_i$ vs freq (%)	43.0	11.0

3. Supplementary Methods

3.1 Automatic parameter optimization

After the introduction of the new I_{CaL} formulation and the achieved physiological inverse dependence between the extracellular Ca^{2+} concentration ($[Ca^{2+}]_o$) and action potential duration (APD), an automatic optimization (similar to (12)) was performed to tune the model in order to fit the experimental data for APD rate dependence and restitution from (5), without losing the correct APD-$[Ca^{2+}]_o$ relationship.

The cost function of the optimization procedure was based on quantitative APD data (5) from different protocols: i) rate dependence at cycle lengths CL = 400, 1,000, 2000 ms; ii) S1S2 restitution with diastolic intervals DI = 10, 20, 30, 40, 50, 70, 100, 150, 200, 500 ms; iii) maximum AP (Vmax); iv) the resting potential (Vrest) and v) the maximum upstroke velocity (dV/dt_{max}). The APD_{90} value for CL=1000ms, at different extracellular Ca^{2+} concentrations ($[Ca^{2+}]_o$ = 1.2, 1.8, 2.4 mM), was used as constrain adding to the cost function the factors $diff_1$ and $diff_2$ to impose the correct inverse APD-$[Ca^{2+}]_o$ relationship.

During the optimization the model was paced for 100 beats in each iteration. The final values of the state variables in each iteration were taken as initial values for the next one. Simulations with the optimized parameters, to quantify the goodness of fit, were run for 1000s to completely ensure steady-state achievement.

3.2 Cost function

In our optimization procedure, we minimized the cost function

$$fcost = \sum_i {\delta_i}^2 + {diff_1}^2 + {diff_2}^2$$

where each term is formulated as:

$$\delta_i = \frac{|Features_{i,Exp} - Features_{i,Sim}|}{Features_{i,Exp}} * weight_i$$

$$diff_1 = \frac{1}{APD_{90}(Cao = 1.8mM) - APD_{90}(Cao = 1.2mM)}$$

$$diff_2 = \frac{1}{APD_{90}(Cao = 2.4mM) - APD_{90}(Cao = 1.8mM)}$$

The parameters chosen for automatic optimization are multiplicative factors for the following current: I_{Kr} (bGKr for the conductance), I_{K1} (bGK1 for the conductance and kslope_IK1 for the steady state rectification slope), I_{NaCa} (bGncx for the conductance), J_{up} (cJup for the conductance), I_{NaL} (bINaL for conductance), J_{diff} (τJdiff for the diffusion time constant), I_{Cab} (gICab for the conductance), I_{CaL} (cPCa for conductance) and kCDI.

We set $weight_i = [0.2\ 0.3]$ for Vmax and dV/dt_{max} respectively, and $weight_i = \sqrt{3}$ for APD_{90}s at CL = 400, 1,000, 2,000 ms in the rate dependence protocols.

3.3 Search method and stop criterion

The initial values of the selected parameters were obtained from a previous modified version of the O'Hara-Rudy model (13) and used to start the automatic optimization.

The minimization of the cost function $fcost$ was based on the trust region reflective algorithm, using the Matlab built-in *lsqnonlin* function (14). The automatic optimization stopped when the number of iteration reached 150 or the minimum change in variables for finite-difference gradients was smaller than 0.1.

Table S3. Initial values and ranges for the optimized parameters

Parameters	Initial	Range [min, max]	Optimal
bGKr	1	[0.1 3]	1.2
bGK1	1	[0.1 3]	0.71
kslope_IK1	1	[0.5 2]	1.09
bGncx	1.2	[0.1 3]	2.4
cJup	10	[1 10]	3.13
bINaL	1	[0.1 3]	2.8
τJdiff	0.2	[0.1 5]	1.9
gICab	1	[0.1 3]	2.3
cPCa	1	[0.65 1.2]	0.9
kCDI	10	[2 10]	9

3.4 Model equations

We present the equations we changed in the BPS2020 with respect to the ORd model. For I_{Kr} the equation was taken from (10) with the adjustment of the conductance (as reported in section 2.1.6) and for I_{NaF} from (15) with the modification presented in section 2.1.10. The equations follow the naming convention of the ORd model. Membrane potential is reported as v.

3.4.1 BPS2020 Human Model Basic Parameters

3.4.1.1 Stimulus

$$amplitude = -53\frac{\mu A}{\mu F}\ , duration = 1\ ms$$

3.4.1.2 Extracellular Concentrations

$$[Na^{+}]_{o} = 144\ mM$$

$$[Ca^{2+}]_{o} = 1.8\ mM$$

$$[\boldsymbol{K}^{+}]_{\boldsymbol{o}} = \begin{cases} \boldsymbol{4\ mM,\ \ if\ experimentally\ used} \\ \boldsymbol{5.4\ mM,\ \ otherwise} \end{cases}$$

3.4.1.3 Cell Geometry

Sarcoplasmic Reticulum Volume

$v_{sr} = 0.95 \cdot (v_{nsr} + v_{jsr})$, with v_{nsr}, v_{jsr} same as ORd model.

3.4.2 BPS2020 Model Initial Conditions

Single endocardial cell, at 1Hz steady state, in diastole with $[K^{+}]_{o}$ = 4mM.

V = -95.6 mV
$[Na^{+}]_{i}$ = 6.5 mM
$[Na^{+}]_{ss}$ = 6.5 mM
$[K^{+}]_{i}$ = 145.4 mM
$[K^{+}]_{ss}$ = 145.4 mM
$[Ca^{2+}]_{i} = 8.5 \cdot 10^{-5}$ mM
$[Ca^{2+}]_{ss} = 8.2 \cdot 10^{-5}$ mM
$[Ca^{2+}]_{sr}$ = 1.63 mM

m = 0.0034
h_{fast} = 0.9398
h_{slow} = 0.9398
j = 0.9398
$h_{slow,CaMK}$ = 0.8521
j_{CaMK} = 0.9398
$m_{L} = 4.45 \cdot 10^{-5}$
h_{L} = 0.73

$h_{L,CaMK} = 0.48$
$a = 0.001$
$i_{fast} = 0.9995$
$i_{slow} = 0.5895$
$a_{CaMK} = 5.24 \cdot 10^{-4}$
$i_{fast,CaMK} = 0.9995$
$i_{slow,CaMK} = 0.6388$
$O_k = 4.87 \cdot 10^{-10}$
$O_{CaMK} = 5.71 \cdot 10^{-7}$
$nca = 4.86 \cdot 10^{-11}$
$x_{s1} = 0.2762$
$x_{s2} = 2.02 \cdot 10^{-4}$
$x_{k1} = 0.9987$
$CaMK_{trap} = 0.0074$
$I1_k = 2.03 \cdot 10^{-8}$
$I2_k = 2.02 \cdot 10^{-8}$
$C_k = 1$
$I1_{k,CaMK} = 2.99 \cdot 10^{-4}$
$I2_{k,CaMK} = 5.58 \cdot 10^{-8}$
$C_{k,CaMK} = 0.9996$
$I1_{Cak} = 4.07 \cdot 10^{-19}$
$I2_{Cak} = 1.22 \cdot 10^{-18}$
$C_{Cak} = 4.85 \cdot 10^{-11}$
$I1_{Cak,CaMK} = 9.26 \cdot 10^{-15}$
$I2_{Cak,CaMK} = 4.97 \cdot 10^{-15}$
$C_{Cak,CaMK} = 4.85 \cdot 10^{-11}$
$j_{nca} = 1$
$RyR_a = 0.0498$
$RyR_o = 2.48 \cdot 10^{-8}$
$RyR_c = 1$
$RyR_{c,CaMK} = 1$
$C_1 = 5.2 \cdot 10^{-9}$
$C_2 = 2.3 \cdot 10^{-5}$
$IC_1 = 0.99$
$IC_2 = 1.9 \cdot 10^{-5}$
$IO = 1.04 \cdot 10^{-5}$
$O = 3.5 \cdot 10^{-5}$

3.4.3 L-type Calcium Current (I_{CaL})

$$ICaL_{tot} = ICaL_{CaMK} * \phi_{ICaL,CaMK} + ICaL * (1 - \phi_{ICaL,CaMK})\ ;$$

$$\phi_{ICaL,CaMK} = \frac{1}{1 + \frac{K_{m,CaMK}}{CaMK_{active}}}$$ % fraction of L-type Calcium channels phosphorylated by CaMK

$$ICaL = ICaL_{VD} + ICaL_{CD}$$
$$ICaL_{CaMK} = ICaL_{VD,CaMK} + ICaL_{CD,CaMK}$$

$$ICaL_{VD} = P_{Ca} * O_{VD} * \Psi_{Ca}$$
$$ICaL_{VD,CaMK} = P_{Ca,CaMK} * O_{VD,CaMK} * \Psi_{Ca}$$
$$ICaL_{CD} = P_{Ca} * O_{CD} * \Psi_{Ca}$$
$$ICaL_{CD,CaMK} = P_{Ca,CaMK} * O_{CD,CaMK} * \Psi_{Ca}$$

3.4.3.1 Driving Force

$$\Psi_{Ca} = z_{Ca}^2 * \frac{VF^2}{RT} * \frac{\gamma_{Cai} * [Ca^{2+}]_{ss} * e^{\frac{z_{Ca}VF}{RT}} - \gamma_{Cao} * [Ca^{2+}]_o}{e^{\frac{z_{Ca}VF}{RT}} - 1}$$
$z_{Ca} = 2$
$\gamma_{Cai} = 1.2$
$\gamma_{Cao} = 0.341$

$$P_{Ca} = \begin{cases} 0.9e^{-3} & endo \\ P_{Ca_{endo}} * 1.4 & epi \\ P_{Ca_{endo}} * 2 & M \end{cases}$$

$$P_{Ca,CaMK} = 1.1 * P_{Ca}$$

3.4.3.2 Markov Model: VDI states

$$\frac{dO_{VD}}{dt} = \alpha * C_{VD} + \delta_{VD} * I1_{VD} - (\beta + \gamma_{VD}) * O_{VD} - r_{up} * O_{VD} + r_{down} * O_{CD}$$

$$\frac{dI2_{VD}}{dt} = \eta_{VD} * I1_{VD} + \omega_{VD} * C_{VD} - (\theta_{VD} + \Psi_{VD}) * I2_{VD} - r_{up} * I2_{VD} + r_{down} * I2_{CD}$$

$$\frac{dI1_{VD}}{dt} = \theta_{VD} * I2_{VD} + \gamma_{VD} * O_{VD} - (\eta_{VD} + \delta_{VD}) * I1_{VD} - r_{up} * I1_{VD} + r_{down} * I1_{CD}$$

$$\frac{dC_{VD}}{dt} = \beta * O_{VD} + \Psi_{VD} * I2_{VD} - (\omega_{VD} + \alpha) * C_{VD} - r_{up} * C_{VD} + r_{down} * C_{CD}$$

$$\frac{dO_{VD,CaMK}}{dt} = \alpha * C_{VD,CaMK} + \delta_{VD,CaMK} * I1_{VD,CaMK} - \left(\beta + \gamma_{VD,CaMK}\right) * O_{VD,CaMK} - r_{up} * O_{VD,CaMK} + r_{down} * O_{CD,CaMK}$$

$$\frac{dI2_{VD,CaMK}}{dt} = \eta_{VD,CaMK} * I1_{VD,CaMK} + \omega_{VD,CaMK} * C_{VD,CaMK} - \left(\theta_{VD,CaMK} + \Psi_{VD,CaMK}\right) * I2_{VD,CaMK} - r_{up} * I2_{VD,CaMK} + r_{down} * I2_{CD,CaMK}$$

$$\frac{dI1_{VD,CaMK}}{dt} = \theta_{VD,CaMK} * I2_{VD,CaMK} + \gamma_{VD,CaMK} * O_{VD,CaMK} - \left(\eta_{VD,CaMK} + \delta_{VD,CaMK}\right) * I1_{VD,CaMK} - r_{up} * I1_{VD,CaMK} + r_{down} * I1_{CD,CaMK}$$

$$\frac{dC_{VD,CaMK}}{dt} = \beta * O_{VD,CaMK} + \Psi_{VD,CaMK} * I2_{VD,CaMK} - \left(\omega_{VD,CaMK} + \alpha\right) * C_{VD,CaMK} - r_{up} * C_{VD,CaMK} + r_{down} * C_{CD,CaMK}$$

3.4.3.3 Markov Model: CDI states

$$\frac{dI2_{CD}}{dt} = \eta_{CD} * I1_{CD} + \omega_{CD} * C_{CD} - (\theta_{CD} + \Psi_{CD}) * I2_{CD} + r_{up} * I2_{VD} - r_{down} * I2_{CD}$$

$$\frac{dI1_{CD}}{dt} = \theta_{CD} * I2_{CD} + \gamma_{CD} * O_{CD} - (\eta_{CD} + \delta_{CD}) * I1_{CD} + r_{up} * I1_{VD} - r_{down} * I1_{CD}$$

$$\frac{dC_{CD}}{dt} = \beta * O_{CD} + \Psi_{CD} * I2_{CD} - (\omega_{CD} + \alpha) * C_{CD} + r_{up} * C_{VD} - r_{down} * C_{CD}$$

$$O_{CD} = 1 - C_{CD} - I1_{CD} - I2_{CD} - C_{VDI} - I1_{VDI} - I2_{VDI} - O_{VDI}$$

$$\frac{dI2_{CD,CaMK}}{dt} = \eta_{CD,CaMK} * I1_{CD,CaMK} + \omega_{CD,CaMK} * C_{CD,CaMK} - \left(\theta_{CD,CaMK} + \Psi_{CD,CaMK}\right) * I2_{CD,CaMK} + r_{up} * I2_{VD,CaMK} - r_{down} * I2_{CD,CaMK}$$

$$\frac{dI1_{CD,CaMK}}{dt} = \theta_{CD,CaMK} * I2_{CD,CaMK} + \gamma_{CD,CaMK} * O_{CD,CaMK} - \left(\eta_{CD,CaMK} + \delta_{CD,CaMK}\right) * I1_{CD,CaMK} + r_{up} * I1_{VD,CaMK} - r_{down} * I1_{CD,CaMK}$$

$$\frac{dC_{CD,CaMK}}{dt} = \beta * O_{CD,CaMK} + \Psi_{CD,CaMK} * I2_{CD,CaMK} - \left(\omega_{CD,CaMK} + \alpha\right) * C_{CD,CaMK} + r_{up} * C_{VD,CaMK} - r_{down} * C_{CD,CaMK}$$

$$O_{CD,CaMK} = 1 - C_{CD,CaMK} - I1_{CD,CaMK} - I2_{CD,CaMK} - C_{VDI,CaMK} - I1_{VDI,CaMK} - I2_{VDI,CaMK} - O_{VDI,CaMK}$$

$$r_{down} = 0.1$$

$$r_{up} = r_{down} * \frac{n}{1-n}$$

$$\frac{dn}{dt} = \alpha_n * k_{2,n} - n * k_{-2,n}, \qquad k_{2,n} = 1000, \qquad k_{-2,n} = 150 * j_n$$

$$\alpha_n = \frac{1-n}{(1+\frac{K_{m,n}}{[Ca]_{SS}})^4}, \qquad K_{m,n} = 0.05$$

$$\frac{dj_n}{dt} = \frac{j_{n\infty} - j_n}{tj_n} \qquad tj_n = 1$$

$$j_{n\infty} = \frac{1}{(1 + e^{\frac{(v+19.58+25)}{3.696}})}$$

3.4.3.4 $\boldsymbol{\alpha/\beta}$ rates ($\boldsymbol{C} \leftrightarrow \boldsymbol{O}$)

$$\alpha = \frac{d_\infty}{\tau_d}$$

$$\beta = \frac{1 - d_\infty}{\tau_d}$$

$$d_\infty = \frac{1}{(1 + e^{\frac{-(v+3.940)}{4.230}})}$$

$$\tau_d = (0.6 + \frac{1}{(e^{(-0.05*(v+6))} + e^{(0.09*(v+14))})}$$

3.4.3.5 $\boldsymbol{\Psi/\omega}$ rates ($\mathbf{I_2} \leftrightarrow \mathbf{C}$)

$$\Psi = \frac{f_\infty}{\tau_{fca}}$$

$$\omega = \frac{1-f_\infty}{\tau_{fca}}$$

$$f_\infty = \frac{1}{(1+e^{\frac{(v+19.58)}{3.969}})}$$

$$\tau_{fca} = 35 + 350 * e^{(\frac{-(v-(-20))^2}{2*10^2})}$$

3.4.3.6 $\boldsymbol{\gamma/\delta}$ rates ($\mathbf{O} \leftrightarrow \mathbf{I_1}$)

$$\gamma_{VD} = \frac{1-f1_{\infty,0}}{\tau_{f1,0}}$$

$$\gamma_{VD,CaMK} = \frac{\gamma_{VD}}{k_{\tau,CaMK}}$$

$$\gamma_{CD} = \gamma_{VD} * kCDI$$

$$\gamma_{CD,CaMK} = \gamma_{VD} * kCDI$$

$$\delta_{VD} = \frac{f1_{\infty,0}}{\tau_{f1,0}}$$

$$\delta_{VD,CaMK} = \frac{\delta_{VD}}{k_{\tau,CaMK}}$$

$$\delta_{CD} = \delta_{VD} * kCDI$$

$$\delta_{CD,CaMK} = \delta_{VD,CaMK} * kCDI$$

$$f1_{\infty,0} = \frac{0.8}{(1+e^{\frac{(v+19.58)}{3.696}})} + 0.2$$

$$\tau_{f1,0} = (70 + \frac{1.2}{\left(0.0045 * e^{\frac{(v+20)}{-50}} + 0.0045 * e^{\frac{(v+30)}{10}}\right)})$$

$$k_{\tau,CaMK} = 2.5$$

$$kCDI = 9$$

3.4.3.7 **θ/η** rates (**I$_1$** ↔ **I$_2$**)

$$\theta_{VD} = \frac{\alpha * \gamma_{VD} * \Psi}{\tau_{f2VD} * (\alpha * \gamma_{VD} * \Psi + \beta * \delta_{VD} * \omega)}$$

$$\theta_{VD,CaMK} = \frac{\alpha * \gamma_{VD,CaMK} * \Psi}{\tau_{f2VD,CaMK} * (\alpha * \gamma_{VD,CaMK} * \Psi + \beta * \delta_{VD,CaMK} * \omega)}$$

$$\theta_{CD} = \frac{\alpha * \gamma_{CD} * \Psi}{\tau_{f2CD} * (\alpha * \gamma_{CD} * \Psi + \beta * \delta_{CD} * \omega)}$$

$$\theta_{CD,CaMK} = \frac{\alpha * \gamma_{CD,CaMK} * \Psi}{\tau_{f2CD,CaMK} * (\alpha * \gamma_{CD,CaMK} * \Psi + \beta * \delta_{CD,CaMK} * \omega)}$$

$$\eta_{VD} = \frac{1}{\tau_{f2VD}} - \theta_{VD}$$

$$\eta_{VD,CaMK} = \frac{1}{\tau_{f2VD,CaMK}} - \theta_{VD,CaMK}$$

$$\eta_{CD} = \frac{1}{\tau_{f2CD}} - \theta_{CD}$$

$$\eta_{CD,CaMK} = \frac{1}{\tau_{f2CD,CaMK}} - \theta_{CD,CaMK}$$

$$\tau_{f2VD} = 100$$

$$\tau_{f2CD} = \frac{\tau_{f2VD}}{kCDI}$$

$$\tau_{f2VD,CaMK} = \tau_{f2VD} * k_{\tau,CaMK}$$

$$\tau_{f2CD,CaMK} = \tau_{f2CD} * k_{\tau,CaMK}$$

3.4.4 SR Calcium Release Flux, via Ryanodine Receptor (J_{rel})

$$J_{rel} = \left(1 - \phi_{rel,CaMK}\right) * J_{rel,NP} + \phi_{rel,CaMK} * J_{rel,CaMK}$$

$$J_{rel,NP} = grel_{max} * RyR_{CaSR} * RyR_o * RyR_c * (Ca_{sr} - Ca_{ss})$$

$$J_{rel,CaMK} = grel_{max,CaMK} * RyR_{CaSR} * RyR_o * RyR_{c,CaMK} * (Ca_{sr} - Ca_{ss})$$

$$\phi_{rel,CaMK} = \frac{1}{1 + \frac{K_{m,CaMK}}{CaMK_{active}}}$$

$$RyR_{CaSR} = 1 - \frac{1}{1 + e^{\frac{Ca_{SR}-0.3}{0.1}}}$$

$RyR_{a1} = 0.05$ (μM), $RyR_{a2} = 0.03$ (μM), $RyR_{a,half} = 0.043$ (μM)

$$RyR_{a\infty} = RyR_{a1} - \frac{RyR_{a2}}{1 + e^{\frac{1000*Ca_{ss}-RyR_{a,half}}{0.0082}}}$$

$$\frac{dRyR_a}{dt} = \frac{RyR_{a\infty} - RyR_a}{\tau_{RyRa}}$$

$$\tau_{RyRa} = 1000\ (ms)$$

$RyR_{o,half} = 0.085$ (μM)

$$RyR_{o\infty} = 1 - \frac{1}{1 + e^{\frac{1000*Ca_{ss}-(RyR_a+RyR_{o,half})}{0.003}}}$$

$$\tau_{RyRo} = 1\ (ms)$$

$$\frac{dRyR_o}{dt} = \frac{RyR_{o\infty} - RyR_o}{\tau_{RyRo}}$$

$RyR_{c,half} = 0.065$ (μM)

$$RyR_{c\infty} = \frac{1}{1 + e^{\frac{1000*Ca_{ss}-(RyR_a+RyR_{c,half})}{0.001}}}$$

$$\tau_{RyRc} = 17.5\ (ms)$$

$$\frac{dRyR_c}{dt} = \frac{RyR_{c\infty} - RyR_c}{\tau_{RyRc}}$$

$$grel_{max} = 0.02$$

$$grel_{max,CaMK} = grel_{max} * 1.25$$

$$\tau_{RyRc,CaMK} = \tau_{RyRc} * 1.25\ (ms)$$

$$\frac{dRyR_{c,CaMK}}{dt} = \frac{RyR_{c\infty} - RyR_{c,CaMK}}{\tau_{RyRc,CaMK}}$$

3.4.5 BPS2020 Human Model Concentrations and Buffers

In all the equations of the concentration balances $v_{nsr}\ or\ v_{jsr}$ have been substituted with v_{sr}.

$$\frac{d[Ca^{2+}]_{sr}}{dt} = \beta_{Casr} \cdot (J_{up} - J_{rel})$$

$$\beta_{Casr} = \frac{1}{1+\frac{\overline{[CSQN]}\cdot K_{m,CSQN}}{(K_{m,CSQN}+[Ca^{2+}]_{sr})^2}},\ \overline{[CSQN]} = 1mM$$

References

1. Leitch S. Effect of Raised Extracellular Calcium on Characteristics of the Guinea-pig Ventricular Action Potential. J Mol Cell Cardiol. 1996 Mar;28(3):541–51.

2. Severi S, Corsi C, Cerbai E. From in vivo plasma composition to in vitro cardiac electrophysiology and in silico virtual heart: the extracellular calcium enigma. Philos Trans A Math Phys Eng Sci. 2009 Jun;367(1896):2203–23.

3. Nagy N, Acsai K, Kormos A, Sebok Z, Farkas AS, Jost N, et al. I-induced augmentation of the inward rectifier potassium current (IK1) in canine and human ventricular myocardium. Pflugers Arch Eur J Physiol. 2013;465(11):1621–35.

4. Severi S, Grandi E, Pes C, Badiali F, Grandi F, Santoro A. Calcium and potassium changes during haemodialysis alter ventricular repolarization duration: in vivo and in silico analysis. Nephrol Dial Transplant. 2008 Apr;23(4):1378–86.

5. O'Hara T, Virág L, Varró A, Rudy Y. Simulation of the undiseased human cardiac ventricular action potential: Model formulation and experimental validation. PLoS Comput Biol. 2011;7(5).

6. Callewaert G. Excitation-contraction coupling in mammalian cardiac cells. Cardiovascular Research. 1992.

7. Li P, Chen SRW. Molecular basis of Ca2+ activation of the mouse cardiac Ca2+ release channel (ryanodine receptor). J Gen Physiol. 2001;118(1):33–44.

8. Drouin E, Lande G, Charpentier F. Amiodarone reduces transmural heterogeneity of repolarization in the human heart. J Am Coll Cardiol. 1998;32(4):1063–7.

9. Glukhov A V., Fedorov V V., Lou Q, Ravikumar VK, Kalish PW, Schuessler RB, et al. Transmural dispersion of repolarization in failing and nonfailing human ventricle. Circ Res. 2010;106(5):981–91.

10. Dutta S, Chang KC, Beattie KA, Sheng J, Tran PN, Wu WW, et al. Optimization of an in silico cardiac cell model for proarrhythmia risk assessment. Front Physiol. 2017;8(AUG):1–15.

11. Grandi E, Pasqualini FS, Bers DM. A novel computational model of the human ventricular action potential and Ca transient. J Mol Cell Cardiol [Internet]. Elsevier Inc.; 2010;48(1):112–21. Available from: http://dx.doi.org/10.1016/j.yjmcc.2009.09.019

12. Fabbri A, Fantini M, Wilders R, Severi S. Computational analysis of the human sinus node action potential: model development and effects of mutations. J Physiol. 2017;595(7):2365–96.

13. Passini E, Severi S. Computational analysis of extracellular calcium effects on an improved human ventricular action potential model. Comput Cardiol (2010). 2012;39:873–6.

14. Thomas F. Coleman and Yuying Li. An Interior Trust Region Approach for Nonlinear Minimization Subject to Bounds. SIAM J Optim. 1996;6(2):418–45.

15. Passini E, Mincholé A, Coppini R, Cerbai E, Rodriguez B, Severi S, et al. Mechanisms of pro-arrhythmic abnormalities in ventricular repolarisation and anti-arrhythmic therapies in human hypertrophic cardiomyopathy. J Mol Cell Cardiol [Internet]. The Authors; 2015;96:72–81. Available from: http://dx.doi.org/10.1016/j.yjmcc.2015.09.003
